# Theoretical study of KRAS G12C secondary mutations influencing inhibitor resistance

**DOI:** 10.2142/biophysico.bppb-v23.0021

**Published:** 2026-07-09

**Authors:** Riksa Meidy Karim, Kazutomo Kawaguchi, Hidemi Nagao

**Affiliations:** 1 Graduate School of Natural Science and Technology, Kanazawa University, Kanazawa, Ishikawa 920-1192, Japan

**Keywords:** drug resistance, molecular dynamics, binding free energy, protein–ligand interaction, KRAS G12C

## Abstract

KRAS is a small GTPase essential for cell signaling, and the G12C mutation acts as a key oncogenic driver in multiple cancers. First-generation KRAS G12C inhibitors, such as sotorasib and adagrasib, have shown clinical efficacy, but are limited by acquired resistance due to secondary mutations. In this study, we investigated the impact of secondary mutations (Y96D, Y96S, G13D, and Q99L) on the binding efficacy of sotorasib, adagrasib, and the next-generation inhibitor (MK-1084) using molecular dynamics simulations, binding free energy calculations, and dynamic protein-ligand interaction analysis. Our study revealed that each secondary mutant variant exhibited variations in the degree of resistance to the inhibitors. Two major resistance patterns were identified: direct and indirect. Our analyses revealed that Y96 mutations directly disrupt inhibitor binding, conferring high resistance to all three inhibitors, whereas G13D and Q99L indirectly alter the binding environment by influencing other residues, resulting in variable resistance profiles. This study provides detailed molecular insights into resistance mechanisms to support the rational design of more robust KRAS G12C-targeted therapies.

## Significance

This study elucidates the structural and energetic mechanisms underlying drug resistance in KRAS G12C mutants. Our findings provided key physical insight into how individual amino acid substitutions contribute to inhibitor resistance. Structural and energetic analyses revealed that mutations act through two mechanisms: a direct mechanism, where the substituted residue disrupts protein–ligand binding and strongly reduces inhibitor efficacy, and an indirect mechanism, where the mutation alters the contributions of neighboring residues in the KRAS binding pocket, producing varying resistance levels. This knowledge can guide the rational design of next-generation inhibitors capable of overcoming acquired resistance in precision oncology.

## Introduction

Kirsten rat sarcoma viral oncogene homologue (KRAS) is a GTPase that serves as a binary molecular switch, transitioning between the active GTP-bound state and inactive GDP-bound state. Its primary role is to facilitate signal transduction in cell proliferation and survival pathways. A specific mutation at codon 12, where glycine (Gly) is substituted with cysteine (Cys), known as KRAS G12C, disrupts the enzyme’s GDP-GTP cycle by locking it in the active GTP-bound configuration. In its active state, KRAS binds to and activates downstream signaling proteins such as RAF. The KRAS G12C mutation profoundly affects the RAF kinase, which is the immediate downstream effector in the RAS-RAF-MEK-ERK signaling cascade (MAPK pathway). The G12C mutation in KRAS hinders its intrinsic ability to hydrolyze GTP to GDP. This effectively traps the KRAS protein in its active, GTP-bound state, causing continuous signaling. The KRAS G12C mutation is prevalent in several types of cancer, including lung, pancreatic, and colorectal cancers [1].

Several drug discovery initiatives have commenced to address the mutation effect of KRAS G12C. Sotorasib (AMG-510) [2] and adagrasib (MRTX849) [3] have emerged as the first-generation inhibitors that successfully target mutant KRAS G12C. However, both clinical and experimental data indicate that these inhibitors can induce resistance due to secondary mutations [4,5]. Consequently, drug resistance has become a significant challenge impeding the advancement of effective treatments in cancer therapies. This situation has shifted the research focus towards addressing the problem of resistance [6].

An *in vitro* study conducted by Koga et al. investigated resistance to sotorasib and adagrasib arising from secondary mutations and identified several key mutants exhibiting significant resistance to each inhibitor [5]. The results indicated that KRAS G12C-Y96D and G12C-Y96S demonstrated strong resistance to both sotorasib and adagrasib. Interestingly, KRAS G12C-Q99L was sensitive to sotorasib but resistant to adagrasib, whereas KRAS G12C-G13D was sensitive to adagrasib but resistant to sotorasib. These findings suggest that each inhibitor exhibited different levels of resistance associated with specific secondary mutations [5]. However, experimental results have limitations in providing a comprehensive molecular description, particularly regarding atomic interactions and the key residues involved in resistance. Identifying these factors is crucial for enhancing the understanding of the fundamental principles and mechanisms underlying resistance.

In addition, next-generation inhibitors of KRAS have been introduced following the initial successes of sotorasib and adagrasib [7]. Among these, Calderasib (MK-1084) is a promising candidate, characterized by its low dosage and favorable bioavailability when administered orally [8]. It demonstrates excellent affinity for the KRAS G12C target, suggesting it is an effective therapeutic option for patients. However, next-generation inhibitors, including MK-1084, are also open to face challenges related to drug resistance due to secondary mutations, similar to their predecessors.

Computational methods, particularly molecular dynamics, provide crucial insights into the thermodynamic properties of inhibitor complexes, elucidating the molecular mechanisms underlying resistance. A range of effective computational methods, including molecular dynamics (MD) simulations and free-energy calculations, are well established for investigating the molecular basis of drug resistance mechanisms caused by protein residue mutations and ligand binding [9–13].

A wide range of computational MD simulations have been conducted to investigate single and double KRAS mutants in both its apo form and inhibitor-bound complexes [14–22]. Comparative analysis derived from molecular dyamics simulation of single mutant (G12C/V/D, G13D, Q61H) and wild type KRAS in apo form shows different mutation exhibits specific dynamics effecting the protein conformation both in active GTP-bound or inactive GDP bound form [14–16]. MD simulation of single mutant KRAS in complex with inhibitor (G12C-Sotorasib, G12D-MRTX1133) has also been previously studied [17–20]. (T. Pantsar, et al) study revealed interaction dynamics between sotorasib and KRAS G12C single mutant via molecular dynamics simulation and showed that sotorasib is stable despite the highly dynamic interface of KRAS’ switches [19]. The results is consistent with (Yu Li, et al) study, with an additional prediction of resistant double mutants (K16A, T35A, Q61A, R68A, H95A, Y96A, Q99A, V103A) using MM/PBSA binding free energy calculation highlighting the possible secondary mutation that could effect the sotorasib binding with KRAS G12C [20]. In the case of the double mutant of KRAS, H. Zhuang et al [21] investigated the mechanistic insight of Y96D secondary mutation in KRAS G12C-Y96D sotorasib complex, discovered the mutation impaired the interaction between the switch II and α3-helix and subsequently induced the opening and loosening of the sotorasib binding pocket. Other double mutant of KRAS G12C were computationally studied by G. Tu, et al in terms of the inhibitor resistance mechanism including the resistance of double mutant G12C-Y96D, G12C-Y96C, G12C-K16T, G12C-R68S to sotorasib and adagrasib [22].

Following the discoveries of the previous studies, we highlighted the effect of specific addition of secondary mutation in KRAS G12C differs according to its inhibitors—mutated/key residues interactions. Experimental results by Koga, et al [5] shows mutant variant G12C-G13D and G12C-Q99L exhibits different levels of resistance to sotorasib and adagrasib. Among the twelve variants examined in that study, we focused specifically on the role of residue G13, a known oncogenic hotspot, as well as the inhibitor-interacting residues Y96 and Q99. Furthermore, we extended our investigation to evaluate the effects of these secondary mutations on the next-generation KRAS G12C inhibitor MK-1084.

This study aimed to investigate the effects of secondary mutation on KRAS G12C inhibitors. We validated the resistance levels of sotorasib and adagrasib by employing molecular dynamics and binding free-energy calculations and comparing the results with experimental data [5] on the mutant variants G12C-Y96D, G12C-Y96S, G12C-G13D, and G12C-Q99L. Through this process, we identified the molecular-level resistance mechanisms via per-residue decomposition analysis and dynamic protein-ligand interactions. Subsequently, we predicted the resistance levels and resistance profile of the next-generation inhibitor MK-1084 using the same methodology. This comprehensive study aims to provide insights into overcoming drug resistance during the ongoing development of KRAS G12C inhibitors.

## Materials and methods

**Figure 1:**
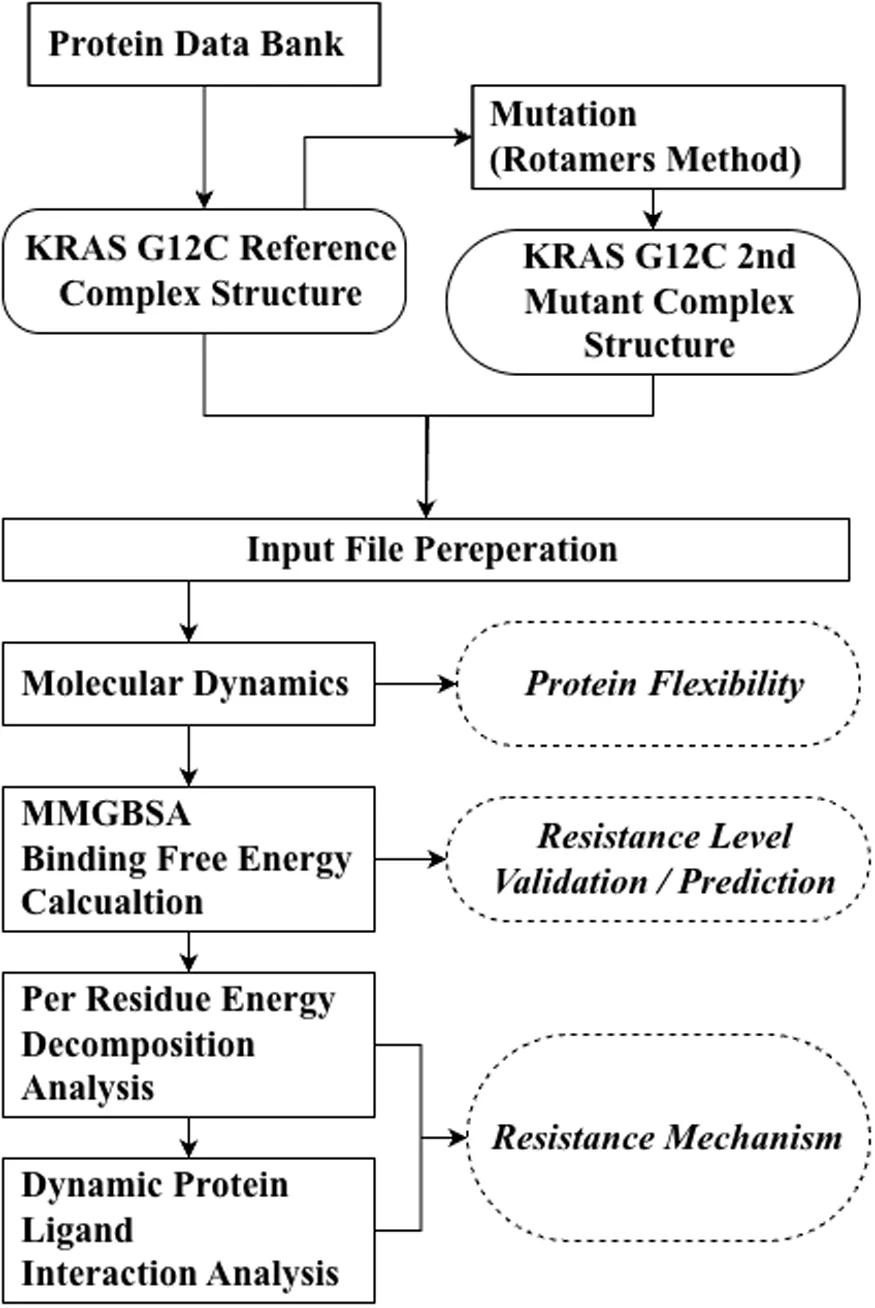
Research Methods

This study investigated a total of 15 distinct KRAS G12C complex structures induced by three inhibitors (sotorasib, adagrasib, MK-1084). Each inihibitor system includes one reference G12C structure and four secondary mutant (G12C-Y96D, G12C-Y96S, G12C-G13D, G12C-Q99L). We utilized various computational methods to explore resistance levels and molecular factors for each inhibitor resulting from four secondary mutations Y96D, Y96S, G13D, and Q99L as outlined in Figure 1. Our approach involved molecular dynamics simulations and MM/GBSA binding free energy calculations for validation and prediction of resistance levels. Additionally, we employed a combination of decomposition analysis and dynamic protein-ligand analyses to describe the mutational effects and profile the resistance mechanisms, as well as to identify key residues contributing to the inhibitor resistance arising from these specific single-point mutations.

### Initial structures

**Figure 2:**
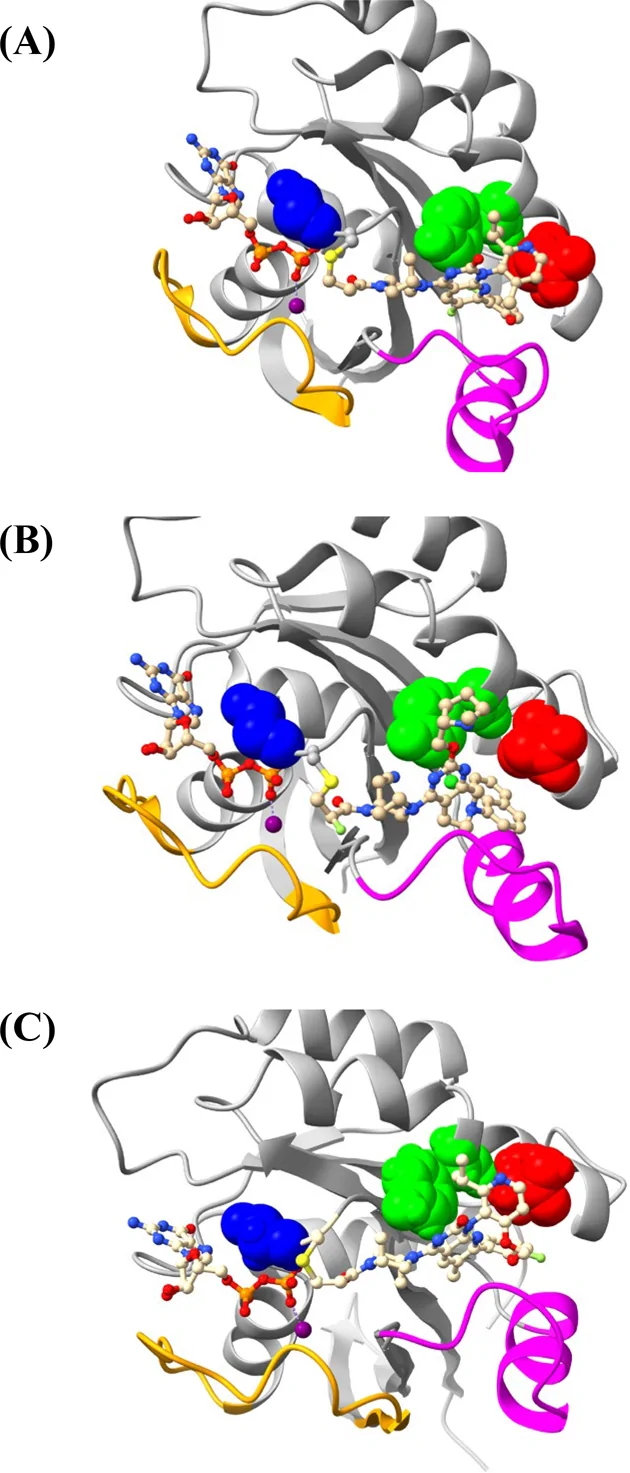
Visualization of the inactive GDP-bound state KRAS G12C in complex with (A) Sotorasib (PDB ID: 6OIM), (B) Adagrasib (PDB ID: 6UT0), (C) MK-1084 inhibitor (PDB ID: 8S8C). The orange color represents Switch I region (residue 25–40). The magenta color represent Switch II region (residue 60–74). The dark purple color represent Mg^2+^ ion. Blue, green, red colors represents the mutated residue Gly13, Tyr96 and Gln99 respectively.

The initial structures of each KRAS G12C reference inhibitor complex were obtained from the Protein Data Bank with the following PDB ID: 6OIM [23] for sotorasib, 6UT0 [3] for adagrasib, and 8S8C [8] for MK-1084 systems. These structures were then visualized using the UCSF ChimeraX software [24] and depicted in Figure 2. We utilized the MODELLER [25] software module to incorporate any missing residues into the models. Thus, we established one reference structure for each inhibitor, which serves as the basis for constructing the secondary mutant systems.

### Construction of secondary mutant systems

The secondary mutant complex structures were derived from the KRAS G12C reference structure by mutating single residues Gly13, Tyr96, or Gln99, using the Dunbrack rotamers library [26] within the ChimeraX rotamers module. Specifically, the G12C-Y96D and G12C-Y96S secondary mutants were generated by replacing residue Tyr96 with Asp96 and Ser96, respectively. The G12C-G13D variant was created by mutating Gly13 to Asp13, whereas the G12C-Q99L was produced by substituting Gln99 with Leu99. The total charge for each ligand was set to 0. Each initial reference system associated with a particular inhibitor yielded four distinct secondary mutant structures.

### Molecular dynamics simulation

The initial preparation of each complex system was performed utilizing CHARMM-GUI Solution Builder [27,28]. The KRAS mutant-inhibitor complex was centered within a rectangular water box, ensuring a buffer distance of 12 Å from the complex to the box edges in all directions. Solvation was performed using the TIP3P water model. To neutralize the system and simulate a physiological ionic strength of 0.15 M, potassium (K^+^) and chloride (Cl^−^) ions were added.

The CHARMM36m force field was utilized for the protein, whereas ligand parameters were derived using the CHARMM General Force Field (CGenFF). The inhibitor coordinates were extracted directly from the crystal structure and submitted for topology and parameter generation. The covalent KRAS G12C–inhibitor complexes were prepared using the PDB Reader module of CHARMM-GUI. During system preparation, the “Add Covalent Bond” option was selected to define the linkage between the sulfur atom of Cys12 and the electrophilic carbon atom of the inhibitor warhead. Covalent attachment occurs through nucleophilic attack of the Cys12 sulfur atom on the β-carbon of the inhibitor acrylamide moiety, yielding a stable thioether adduct following Michael addition [29].

All simulations were performed using GROMACS 2024.2 [30], employing periodic boundary conditions in all dimensions. Long-range electrostatics were calculated using the Particle Mesh Ewald (PME) method, with a real-space cutoff set at 1.2 nm. The Lennard-Jones interactions were gradually switched off between 1.0 and 1.2 nm.

Energy minimization was carried out using the steepest descent algorithm until the system achieved a maximum force of less than 1000 kJ/mol/nm. This was followed by a multi-step equilibration protocol as outlined by CHARMM-GUI, involving the NVT ensemble for 250 ps and the NPT ensemble for 500 ps, with position restraints gradually released. The temperature was maintained at 300 K using the Nosé–Hoover thermostat, and the pressure was maintained at 1 atm using the Parrinello–Rahman barostat.

A production molecular dynamics simulation was conducted in an unrestrained manner over a period of 100 ns within the NPT ensemble, utilizing a time step of 2 femtoseconds. To ensure accuracy, all covalent bonds involving hydrogen atoms were constrained using the LINCS algorithm. Trajectory data was recorded every 100 picoseconds to facilitate comprehensive post-simulation analysis. Post-processing and analysis were performed using GROMACS tools and MDAnalysis python library [31,32]. Structural stability and dynamics [33] were assessed by calculating the root mean square deviation (RMSD) and root mean square fluctuation (RMSF) as follows:


(1) $$RMSD\left(t\right)=\sqrt{\frac{1}{n}\sum_{i=1}^{n}{∥r_{i}\left(t\right)-r_{i}^{ref}∥}^{2}}$$

(2) $$RMSF\left(i\right)=\sqrt{\frac{1}{N}\sum_{i=1}^{N}{∥r_{i}\left(t\right)-\left\langle r_{i}\right\rangle ∥}^{2}}$$

where n is total number of atoms, $r_{i}\left(t\right)$ is the vector position of atom i at time t, $r_{i}^{ref}$ is the position vector of atom i in the reference structure. N is the total number of frames and $\left\langle r_{i}\right\rangle$ is the average position of atom i over the trajectory.

### Binding free energy calculation using MM/GBSA method

The binding free energy between the KRAS mutant and inhibitor was calculated using the Molecular Mechanics/Generalized Born Surface Area (MM/GBSA) method [34], implemented via the gmx_MMPBSA tool [35,36]. This approach combines molecular mechanics energies with solvation free energy estimates to approximate the binding affinity based on the last 10 ns of structural snapshots from the MD trajectory, as described in the following equation:

(3) $$\begin{array}{c} \Delta G=\left\langle G_{C}\right\rangle -\left\langle G_{P}\right\rangle -\left\langle G_{L}\right\rangle \\ \left\langle G_{x}\right\rangle =\left\langle E_{x,MM}\right\rangle +\left\langle G_{x,solv}\right\rangle \\ \Delta H=\Delta E_{MM}+\Delta G_{solv}\\ {\Delta G}_{bind}=\Delta H-T\Delta S \end{array}$$

where ${\Delta G}_{bind}$ is the binding free energy of the KRAS mutant and inhibitor, $G_{C}$, $G_{P}$ and $G_{L}$ represent the Gibbs free energy of the complex, protein, and ligand, respectively. $\left\langle G_{x}\right\rangle$ is the ensemble average of G over N trajectory frame. $\left\langle E_{x,MM}\right\rangle$ denotes the average molecular mechanics energy, including electrostatic and van der Waals interactions. $\left\langle G_{x,solv}\right\rangle$ represents the ensemble average of the solvation free energy consisted of polar and non polar terms. Polar terms were calculated using the Generalized Born model and non polar terms were estimated using the solvent accessible surface area. The entropy was estimated with the interaction entropy (IE) method [37].

To improve conformational sampling and ensure reproducibility, three independent 100 ns MD simulations were performed for each KRAS–ligand complex using different initial velocity distributions. Structural and energetic analyses were subsequently carried out using the equilibrated trajectories obtained from the replicate simulations.

### Per residue binding free energy decomposition analysis

To identify the key amino acid residues involved in ligand binding, a per-residue energy decomposition analysis was also conducted using the gmx_MMPBSA tool. This analysis provides valuable insights into the contributions of individual residues to the overall binding free energy by decomposing the MM/GBSA energy terms. The last 10 ns of the trajectory were analyzed to determine the residue-wise contributions from the protein. The following energy components were computed for each residue:

(4) $$\Delta G_{bind}^{Res}=\Delta G_{ele}^{Res}+\Delta G_{vdW}^{Res}+\Delta G_{solv,p}^{Res}+\Delta G_{solv,p}^{Res}$$

where Res is the corresponding residue, $\Delta G_{ele}^{Res}$ is the electrostatic contribution of residue Res to the inhibitor, $\Delta G_{vdW}^{Res}$ is the van der Waals energy, $\Delta G_{solv,p}^{Res}$ is the polar solvation energy term, and $\Delta G_{solv,p}^{Res}$ is the nonpolar solvation term of residue Res.

### Dynamic protein-ligand interaction analysis

To further characterize the molecular interactions driving the binding affinity, a protein–ligand interaction fingerprint (PLIF) analysis was performed using the ProLIF Python library [38]. ProLIF was used to compute the occurrence frequency of various interaction types, including hydrogen bonds (donor–acceptor pairs), hydrophobic contacts, π–π stacking, cation–π interactions, and van der Waals contacts. For each trajectory frame, a binary fingerprint was generated, marking the presence or absence of each interaction between the ligand and surrounding protein residues. The interaction frequency was then calculated by averaging over all the frames as shown in the following:

(5) $$f_{int}^{Res}=\frac{N_{int}^{Res}}{N}$$

where $f_{int}^{Res}$ is the interaction frequency between the inhibitor and residue Res of the interaction type int, $N_{int}^{Res}$ is the number of contacts, and *N* is the total number of frames.

### Resistance level calculation

We defined the resistance level obtained from our method as the relative binding free energy of KRAS G12C secondary mutant with respect to the KRAS G12C reference as follows:

(6) $$\Delta \Delta G=\Delta G^{mut}-\Delta G^{ref}$$

where ΔΔG is the computed resistance level, $\Delta G^{mut}$is the binding free energy of the inhibitor and KRAS G12C secondary mutant, $\Delta G^{ref}$ is the binding free energy of the KRAS G12C reference.

The resistance level was validated by comparing the results with the experimental data from Koga, et al study [5] as follows:

(7) $${\Delta \Delta G}_{\exp}=RT\ln\left(\frac{{IC}_{50}^{mut}}{{IC}_{50}^{ref}}\right)$$

where ${\Delta \Delta G}_{\exp}$ is the experimental resistance level, ${IC}_{50}^{mut}$ is Half-Maximal Inhibitory Concentration of the secondary KRAS G12C secondary mutant with the inhibitor, and ${IC}_{50}^{ref}$ is the Half-Maximal Inhibitory Concentration of the reference KRAS G12C with the inhibitor.

Thus, we compared our computed ΔΔG resistance level and experimental ${\Delta \Delta G}_{\exp}$ by calculating the coefficient of determination (r^2^) and mean squared error (MSE). The r^2^ value measures the proportion of variance in the experimental relative binding free energies (ΔΔ*G*) that is explained by our computational predictions, with values closer to 1 indicating a strong correlation and better predictive accuracy. The MSE, on the other hand, quantifies the average squared deviation between the computed and experimental ΔΔ*G* values, with lower values reflecting higher precision and closer agreement between the two datasets. These metrics are described by the following equation:

(8) $$r^{2}=1-\frac{\sum_{i=1}^{N}{\left(\Delta \Delta G_{exp,i}-\Delta \Delta G_{i}\right)}^{2}}{\sum_{i=1}^{N}{\left(\Delta \Delta G_{exp,i}-\bar{\Delta \Delta G_{\exp}}\right)}^{2}}$$

(9) $$MSE=\frac{1}{N}\sum_{i=1}^{N}{\left(\Delta \Delta G_{i}-\Delta \Delta G_{exp,i}\right)}^{2}$$

where $\Delta \Delta G_{i}$ is the calculated resistance level of the *i*^th^ system, $\Delta \Delta G_{\exp,i}$ is the experimental resistance level of the *i*^th^ system, *N* is the total number of systems and $\bar{\Delta \Delta G_{\exp}}$ is the mean of the experimental resistance level.

### Identification of key residues and interaction leading to resistance

To identify the key residues influencing resistance, we examined the residue-wise contribution to the resistance level (Equation 10) and tracked the changes in the interaction frequency (Equation 11) between the KRAS G12C reference systems and KRAS G12C secondary mutant systems.

(10) $$\Delta \Delta G^{Res}=\Delta G^{Res,mut}-\Delta G^{Res,ref}$$

$\Delta \Delta G^{Res}$ represents the contribution of residue Res in the resistance level, $\Delta G^{Res,mut}$ is the binding free energy contribution for residue Res with inhibitor in the secondary mutant systems, $\Delta G^{Res,ref}$ is the contribution of residue Res in binding free energy with inhibitor in the KRAS G12C reference systems.

(11) $$\Delta f_{int}^{Res}=f_{int}^{Res,mut}-f_{int}^{Res,ref}$$

$\Delta f_{int}^{Res}$ represents the changes in the interaction frequency of residue Res with type int to the inhibitor from the secondary mutant systems to the reference systems, while $f_{int}^{Res,mut}$ and $f_{int}^{Res,ref}$ represent residue Res interaction frequency of a particular interaction type int in the secondary mutant systems and the reference system, respectively.

## Results and discussion

Our study focused on three key findings: the stability of the KRAS G12C mutant in complex with each inhibitor, the resistance levels exhibited by each inhibitor, and the molecular resistance profile attributed to specific residues within the complex.

### Complex dynamics and stability

Prior to the MD simulations, to validate the structural integrity of the modeled systems, the secondary mutant complexes were superimposed onto the reference KRAS G12C crystal structure (PDB ID: 6OIM, 6UT0, 8S8C), as summarized in Table 1. All mutants, including G12C-Y96D, G12C-Y96S, G12C-G13D, and G12C-Q99L, exhibited low backbone RMSD values (<0.5 Å), indicating high structural similarity to the crystal structure. Minor deviations were primarily associated with local side-chain rotameric rearrangements, whereas the overall secondary structural architecture remained preserved.

**Figure 3:**
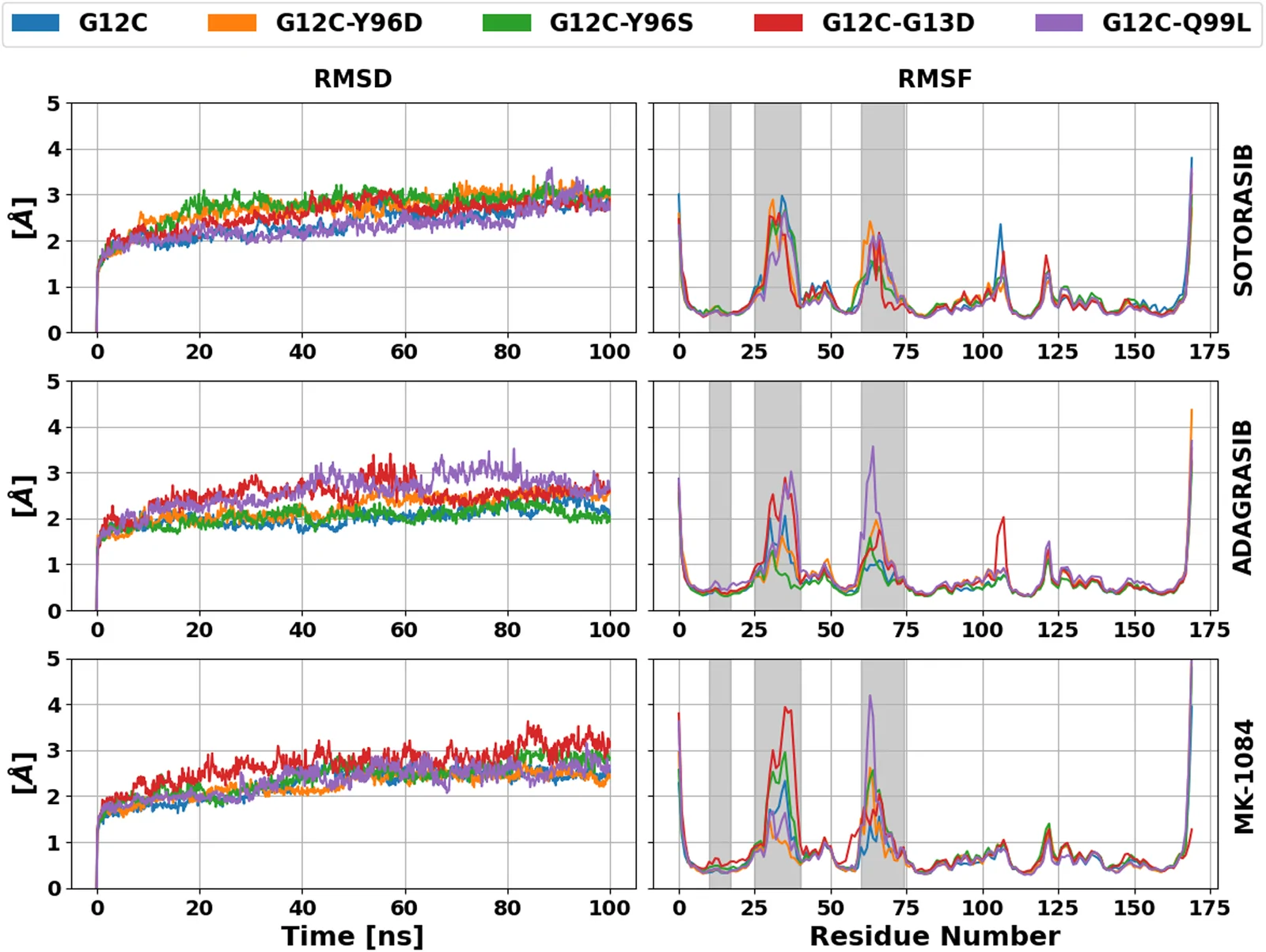
RMSD and RMSF analyses of all 15 KRAS–ligand complexes during the MD simulations. Comparative B-factor analyses with the corresponding crystal structures are provided in Supplementary Figure S3.

Following the initial structure, a 100 ns molecular dynamics simulation was conducted for each complex. The trajectory was subsequently analyzed to assess the overall global stability of each complex through the calculation of the RMSD value. As illustrated in Figure 3, the RMSD values for the protein-ligand complexes across all KRAS-inhibitor systems remained below 4 Å, indicating robust global stability. Nonetheless, despite this overall stability (global RMSD<4 Å), the secondary mutants demonstrated increased flexibility within the Switch I (residues 25–40) and Switch II (residues 60–74) regions. These regions are essential for nucleotide exchange and effector binding. This observation suggests that, while the complexes exhibit a stable conformational framework on a global scale, the presence of secondary mutations influences local dynamics, affecting functional interactions.

**Figure 4:**
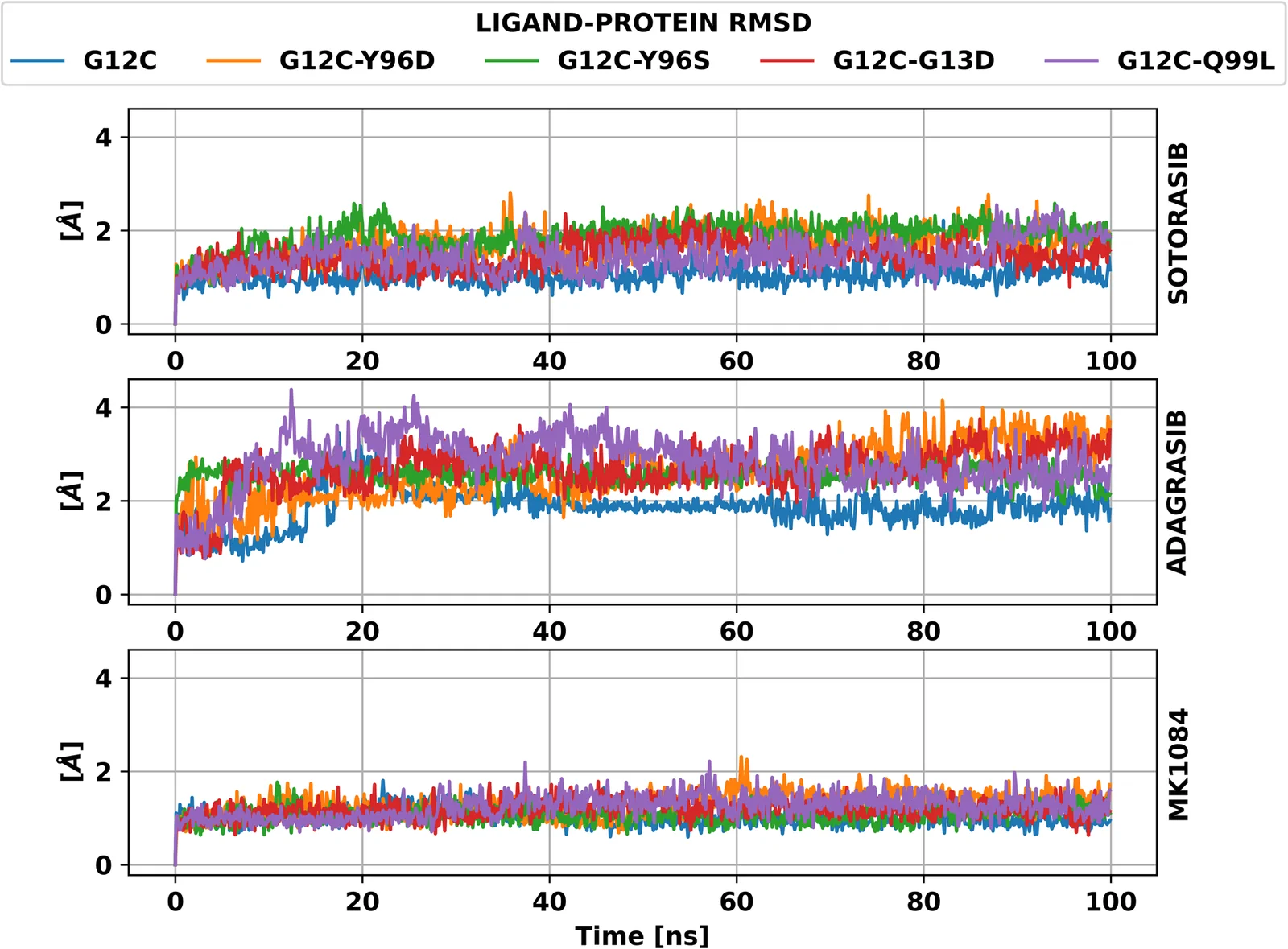
Ligand RMSD analysis of mutant KRAS complexes with Sotorasib, Adagrasib, and MK-1084 throughout the MD simulations. RMSD values were calculated for the ligands after fitting the trajectories to the protein backbone to assess the stability of ligand binding relative to the initial crystal structure (blue) conformation.

To assess ligand stability during the simulations, ligand RMSDs were calculated after fitting the trajectories to the protein backbone atoms only. Stable RMSD values observed for all KRAS–ligand complexes indicated persistent ligand binding throughout the simulation time as shown in Figure 4. MM/GBSA binding free energy calculations were subsequently performed using equilibrated trajectory frames to ensure reliable estimation of ligand binding affinities. In both the G12C-Y96D and G12C-G13D systems, adagrasib exhibits relatively higher ligand RMSD values (>3 Å) with noticeable fluctuations (Supplementary Figures 1 and 2). This instability stems from conformational changes within the KRAS binding pocket induced by the secondary mutations.

### Resistance level validation and prediction

The resistance level of each inhibitor is defined by the relative binding affinities of the secondary mutant systems compared to the reference system. This was evaluated across four KRAS mutants—G12C-Y96D, G12C-Y96S, G12C-G13D, and G12C-Q99L—using ΔΔ*G* values, which reflect the extent of binding disruption. A ΔΔ*G* value exceeding 2.74 kcal/mol indicates substantial disruption, while values below this threshold suggest moderate and lower levels of binding impairment. This threshold was selected based on the resistance index ($\frac{IC_{50}^{mut}}{IC_{50}^{ref}}$) from the experimental data of Koga, et al study [5]. The study used the resistance index (RI) value as the basis to differentiate high resistance (RI>100) with low (RI<10) and moderate resistance (10<RI<100). Based on these thresholds, we converted the RI value to the resistance level threshold (ΔΔ*G*) using Equation 7. We obtained two thresholds ΔΔ*G*=1.37 kcal/mol (RI=10) and ΔΔ*G*=2.74 kcal/mol (RI=100) with T=300K matched with our simulation temperature. Accordingly, we classified the low (ΔΔ*G*<1.37 kcal/mol), moderate (1.37<ΔΔ*G*<2.74 kcal/mol) and high (ΔΔ*G*>2.74 kcal/mol) resistance level using the two obtained thresholds.

**Figure 5:**
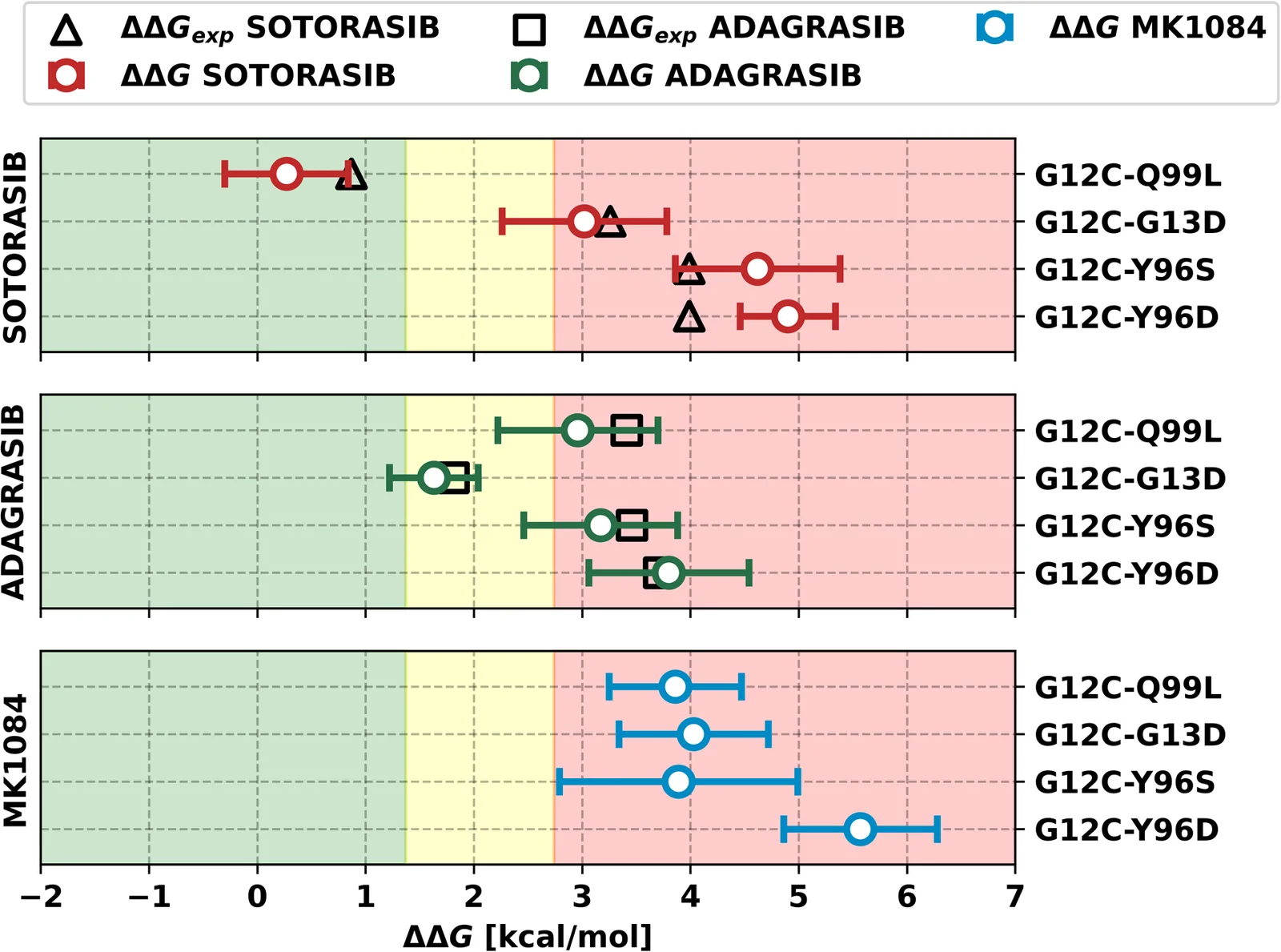
Inhibitor Resistance Level for Sotorasib, Adagrasib and MK-1084 to all KRAS G12C mutants. Green colored area represents Low Resistance, while yellow and red color represent Moderate and High Resistance respectively (Threshold: 1.37 and 2.74 kcal/mol).

**Figure 6:**
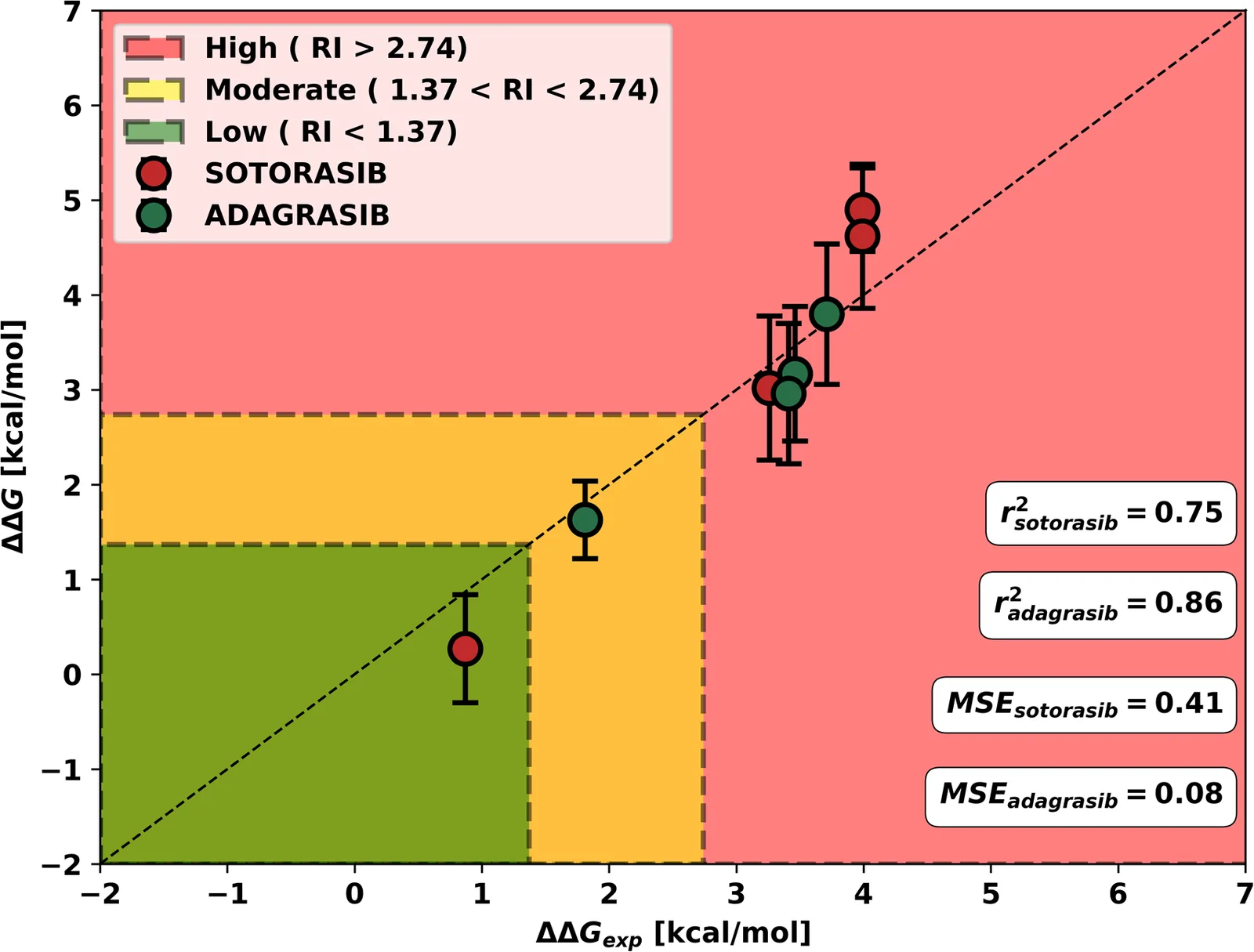
Inhibitor Resistance Level Compared with Experimental Data from Koga, et al [5] study. (Threshold: 1.37 and 2.74 kcal/mol).

Our findings (Figure 5, Table 2) indicate that sotorasib and adagrasib exhibited high resistance to the Y96D/S secondary mutations, whereas adagrasib showed moderate resistance to the G13D secondary mutation, and sotorasib demonstrated low resistance to the Q99L secondary mutation. Given the threshold, our calculated resistance levels were consistent with the experimental data, both for sotorasib and adagrasib inhibitor (Figure 6). Experimentally and computationally, both inhibitors showed high resistance level arising from the secondary mutant Y96D/S. This experimental and computational consistency also held for secondary mutation Q99L and G13D. Sotorasib showed low resistance level for secondary mutant Q99L, but high resistance level for secondary mutant G13D. In contrast, adagrasib inhibitor showed moderate resistance level for secondary mutant G13D, but high resistance level for secondary mutant Q99L. Therefore, we accurately validated the resistance level of sotorasib and adagrasib inhibitor across all four mutant variants.

Sotorasib exhibited the highest degree of binding disruption for the G12C-Y96D (4.90 kcal/mol) and G12C-Y96S (4.62 kcal/mol) mutants, suggesting a significant reduction in affinity. The G12C-G13D mutation (3.02 kcal/mol) also resulted in considerable disruption, whereas Q99L (0.27 kcal/mol) had a minimal effect. Similarly, adagrasib demonstrated high binding disruption for G12C-Y96D (3.80 kcal/mol) and G12C-Y96S (3.17 kcal/mol). However, unlike sotorasib, adagrasib exhibited notable binding impairment with Q99L (2.96 kcal/mol), whereas G12C-G13D (1.63 kcal/mol) showed lower disruption.

A previous study by G. Tu [22] calculated the MM/GBSA binding free energies for the mutant G12C-Y96D, resulting in +10.03 kcal/mol for sotorasib and +10.63 kcal/mol for adagrasib. In comparison, our calculated values (+4.90 and +3.80 kcal/mol respectively) are closer to the experimental data reported by Koga et al. (+3.99 kcal/mol for sotorasib and +3.71kcal/mol for adagrasib). However, when applying the resistance threshold of 2.74 kcal/mol, both studies consistently predict a high level of resistance. These discrepancies in absolute values likely arise from differences in the simulation setups, while G.Tu et al. utilized Amber18 with the ff14SB force field, our simulation was performed using GROMACS with the CHARMM36m force field.

Employing a similar methodology, we assessed the predicted resistance levels of the next-generation MK-1084 inhibitor. Our findings revealed that MK-1084 exhibited a high resistance level to all four identified KRAS secondary mutations, indicating a significant disruption in its ability to bind effectively. In contrast to Sotorasib and Adagrasib, MK-1084 displayed a consistently high degree of binding disruption across all mutants, with the most pronounced effect observed for G12C-Y96D (5.57 kcal/mol), followed by G12C-Y96S (3.89 kcal/mol), G12C-G13D (4.03 kcal/mol), and Q99L (3.86 kcal/mol). Notably, the binding affinity of the KRAS G12C reference mutant for MK-1084 is measured at –85.35 kcal/mol, showcasing a superior affinity in comparison to sotorasib, which has a binding affinity of –76.55 kcal/mol, and adagrasib, which registers at –83.22 kcal/mol. Despite this apparent advantage in binding affinity with the reference mutant, the efficacy of MK-1084 was severely compromised due to the presence of these secondary mutations. The observed high resistance indicates that the long-term effectiveness of MK-1084 is likely diminished, posing challenges for its sustained therapeutic use in patients with KRAS-driven cancers.

The computational predictions for sotorasib and adagrasib demonstrated strong agreement with experimental data (Figure 6), although there were some notable differences. For sotorasib, the r^2^ value of 0.75 indicates that 75% of the variance in the experimental relative binding free energies (ΔΔ*G*) was explained by the computational model, reflecting a moderate to strong correlation between predicted and experimental values. An MSE of 0.41 further confirms the reasonable accuracy, with predictions deviating only slightly from the experimental data. In contrast, for adagrasib, the high r^2^ value of 0.86 suggested a strong correlation, explaining 86% of the variance in the experimental data. Furthermote, the very low MSE of 0.08 indicates that the computational predictions were highly precise, with minimal deviation from the experimental values.

In addition, a previous study by G. Tu [22] calculated the MM/GBSA binding free energy of double mutants (G12C-Y96D, G12C-Y96C, G12C-K16T, and G12C-R68S) bound to sotorasib and adagrasib, yielding r^2^ values of 70.58% and 95.35%, respectively. Compared to those findings, our r^2^ value for sotorasib is 5% higher, whereas our correlation for adagrasib is 9% lower. Nevertheless, we observed the same overall trends, with adagrasib demonstrating a stronger correlation (86% to 95%) than sotorasib (70% to 75%) This discrepancy arises from the different mutants analyzed. While the previous study examined Y96C, K16T, and R68S, our work utilized the Y96S, G13D, and Q99L mutants, with additional next generation inhibitor MK-1084.

Although the calculated ΔΔ*G* values were strongly consistent with experimental data, differences were observed between the Δ*G* values obtained from MM/GBSA and the experimental results. This difference arises from the limitations of MM/GBSA method in obtaining the precise absolute binding free energies compared with more robust methods such as Free Energy Perturbation. However, MM/GBSA provides better results than simple docking scores and is widely used in computer-aided drug discovery to predict binding affinity between proteins and ligands [39]. Another advantage of the MM/GBSA method over Free Energy Perturbation is that it is more computationally efficient. As most drug-discovery studies focus on identifying trends in differences between binding free energies, the respectable accuracy and efficiency of the MM/GBSA method are advantageous for our study, which focuses on identifying relative binding free-energy shifts from secondary to primary mutants. Hence, despite differences in Δ*G* values between MM/GBSA and experimental data, our method remained consistent in predicting relative binding free energy values and the resistance levels as shown in the consistency of the overall trend of the ΔΔ*G* values.

### Identification of key residues and interaction driving resistance

Following the findings regarding the resistance levels of each inhibitor, we identified the key residues and interactions that contribute to this resistance. We employed MM/GBSA binding free energy decomposition analysis at the residue level, along with an analysis of protein-ligand interactions. Based on this analysis, we generated resistance profiles for each inhibitor complex, highlighting the effects of secondary mutation influencing the inhibitor resistance.

**Figure 7:**
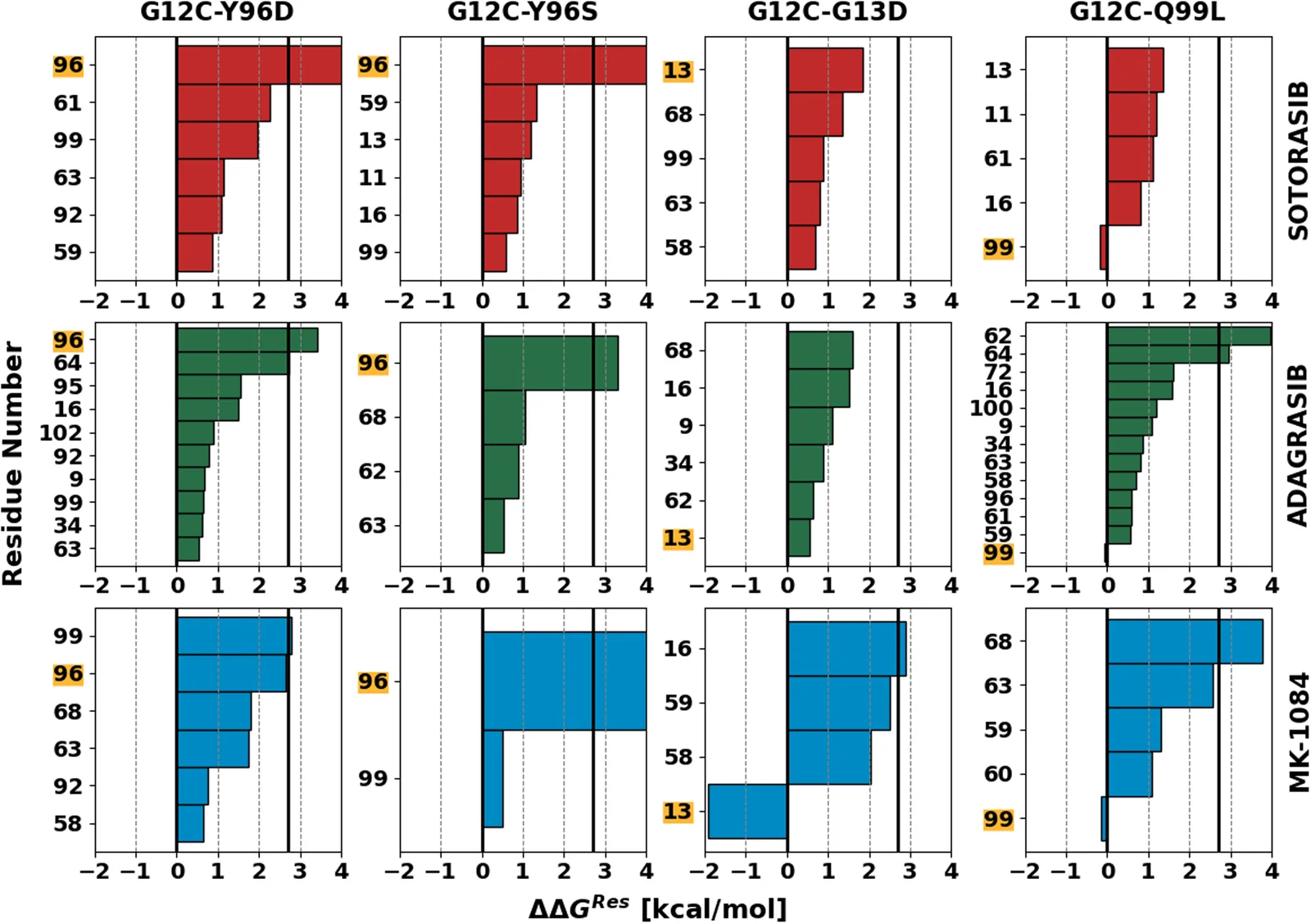
Key Residues and their contribution to resistance. The orange color highlights the mutated residues. Vertical black line represent High Resistance threshold (2.74 kcal/mol)

**Figure 8:**
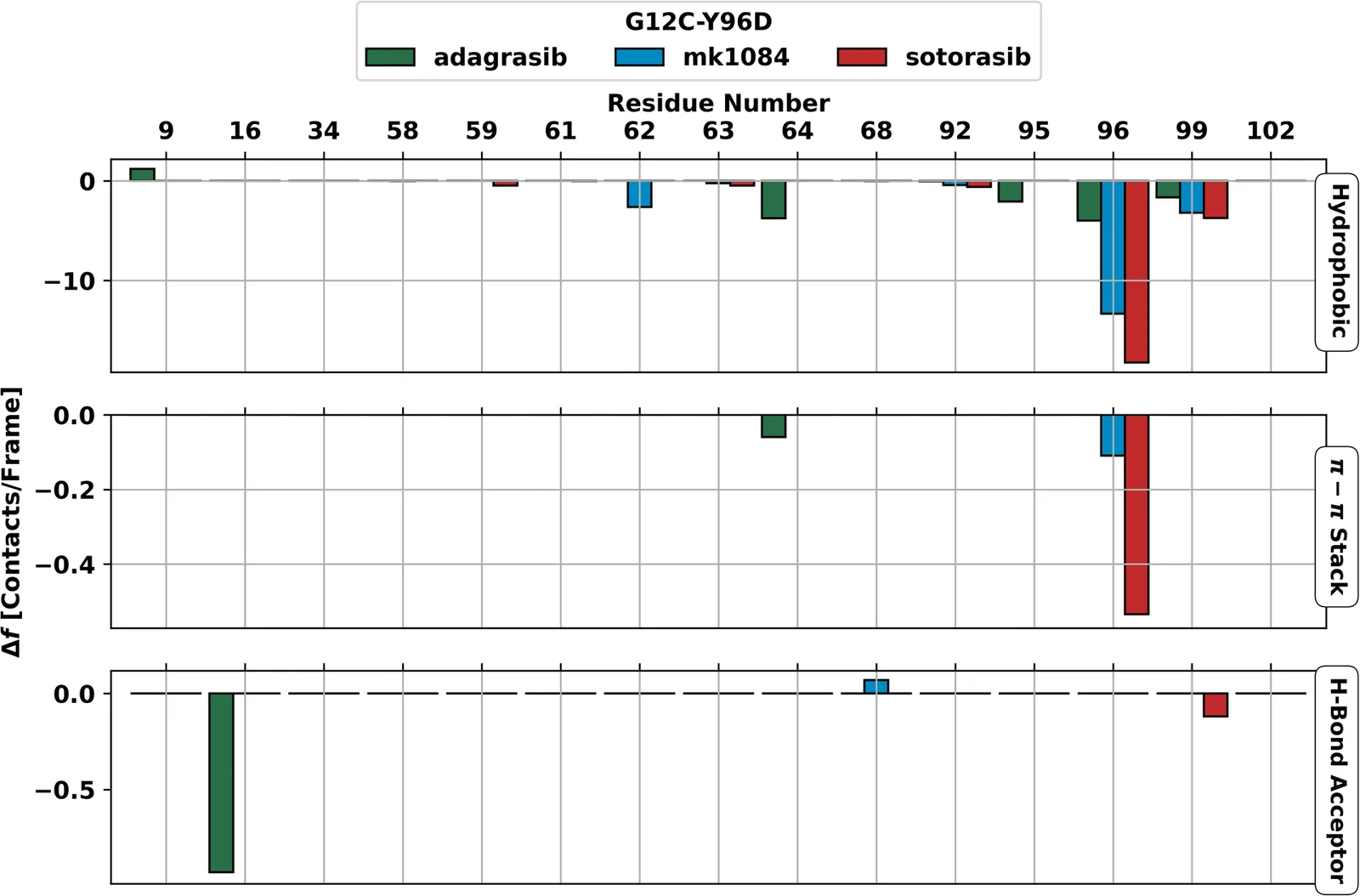
Key residue delta interaction frequency for KRAS G12C-Y96D secondary mutant complex. Detailed interaction frequencies are provided in Supplementary Table S2, while representative binding mode visualizations are shown in Supplementary Figures S4, S1, and S5 for sotorasib, adagrasib and MK-1084 systems respectively.

**Figure 9:**
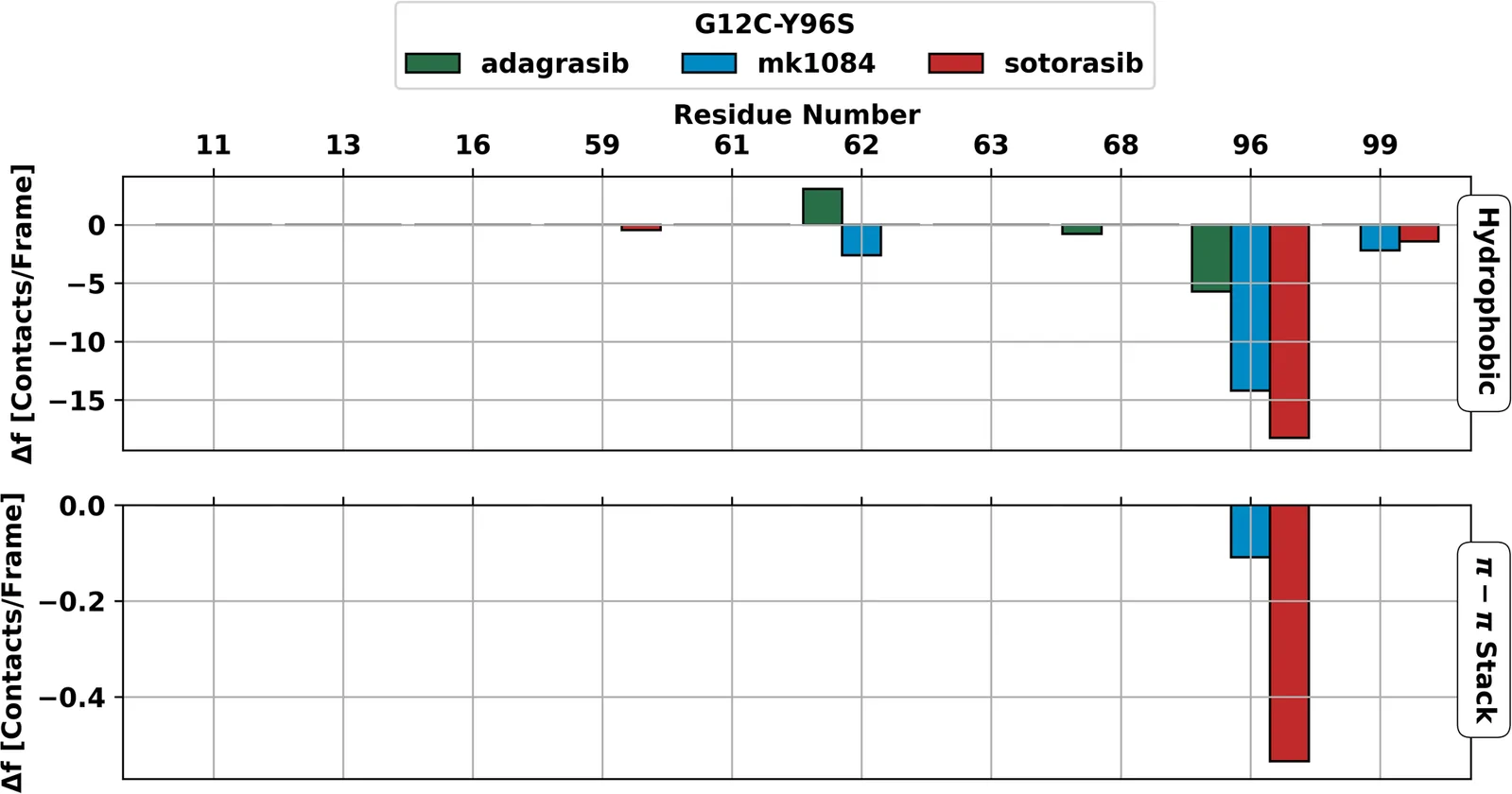
Key residue delta interaction frequency for KRAS G12C-Y96S secondary mutant complex. Detailed interaction frequencies are provided in Supplementary Table S3, while representative binding mode visualizations are shown in Supplementary Figures S6, S7, and S8 for sotorasib, adagrasib and MK-1084 systems respectively.

**Figure 10:**
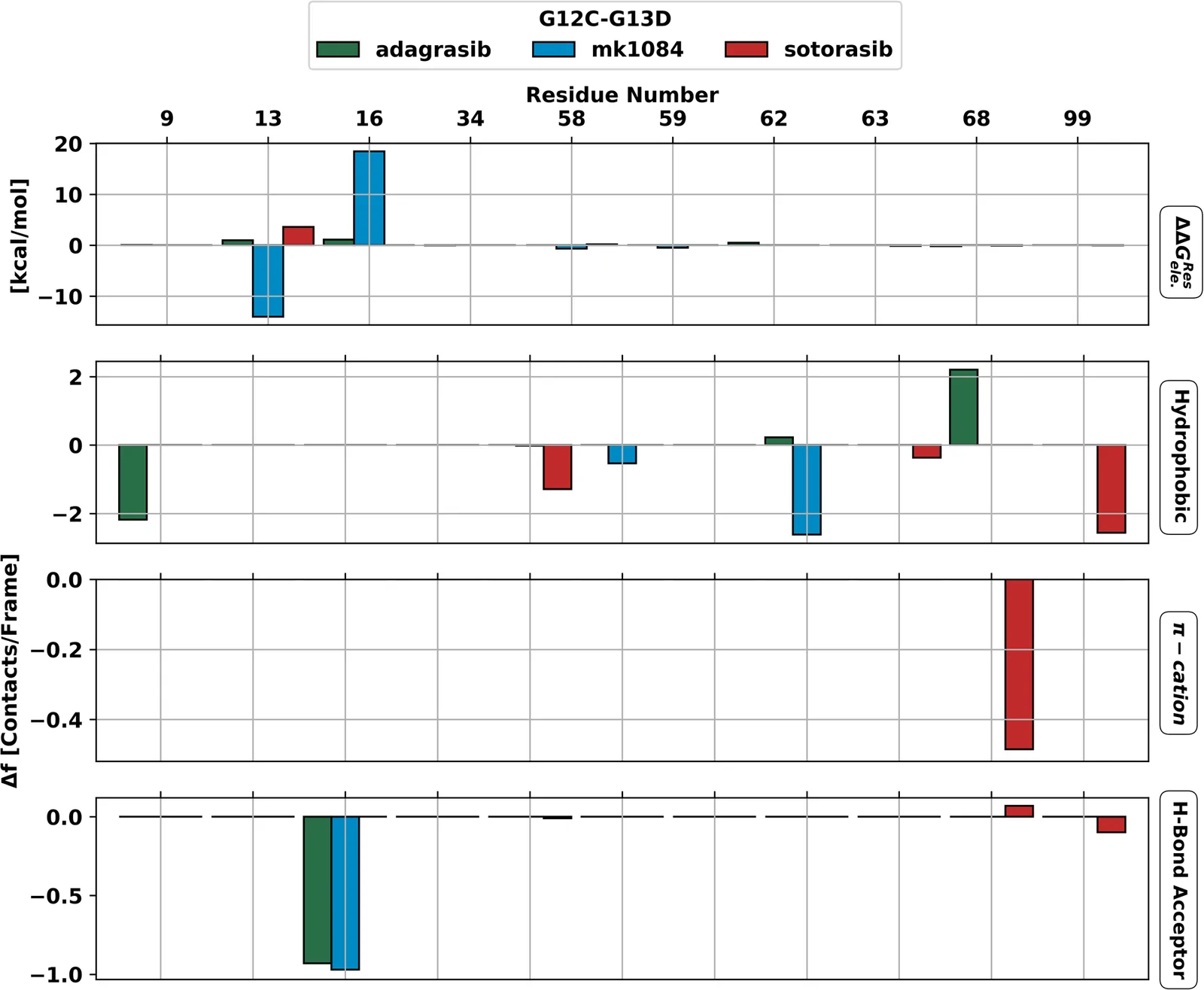
Key residue energy decomposition and delta interaction frequency for KRAS G12C-G13D secondary mutant complex. Detailed interaction frequencies are provided in Supplementary Table S4, while representative binding mode visualizations are shown in Supplementary Figures S9, S2, and S10 for sotorasib, adagrasib and MK-1084 systems respectively.

**Figure 11:**
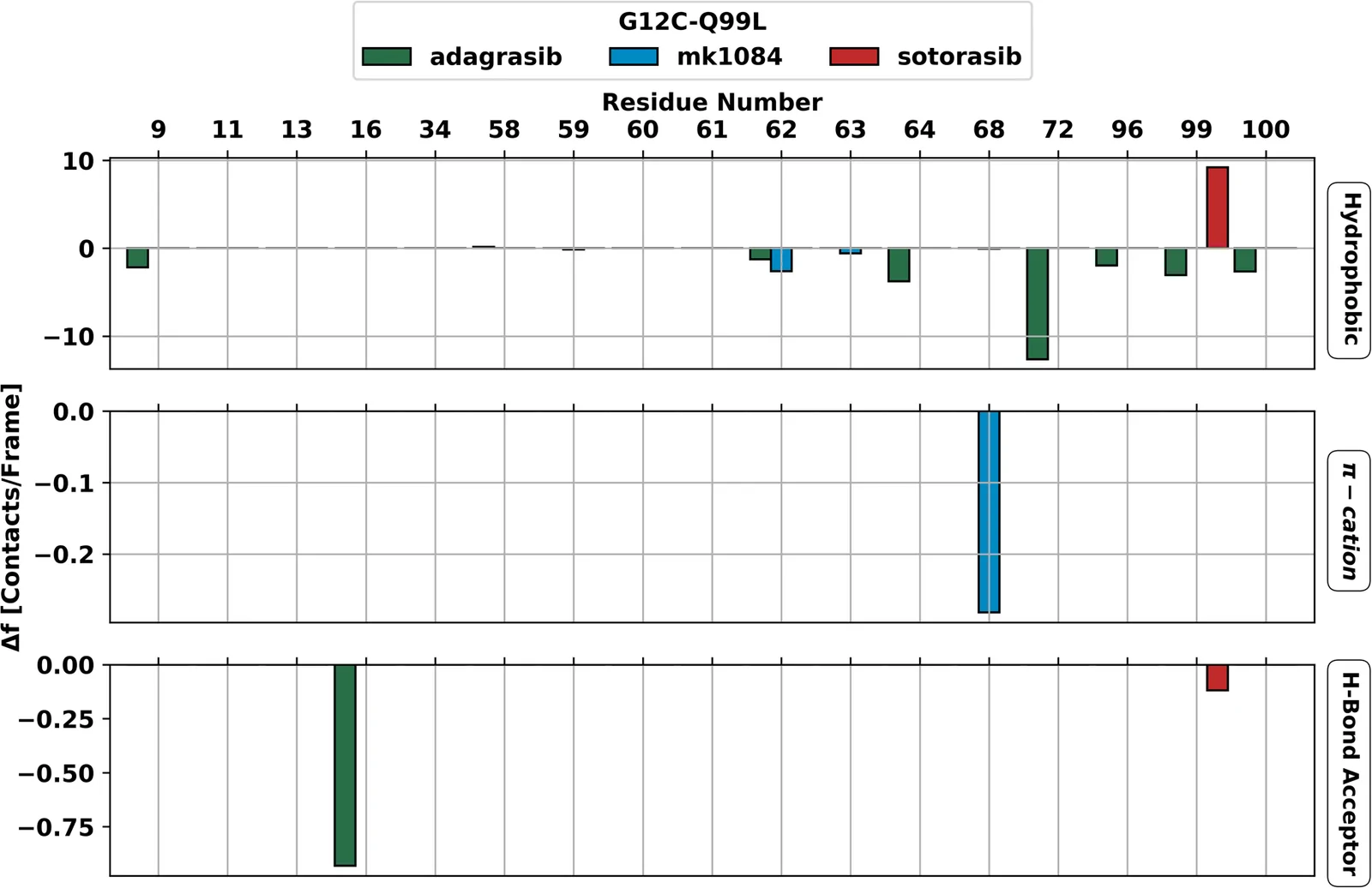
Key residue delta interaction frequency for KRAS G12C-Q99L secondary mutant complex. Detailed interaction frequencies are provided in Supplementary Table S5, while representative binding mode visualizations are shown in Supplementary Figures S11, S12, and S13 for sotorasib, adagrasib and MK-1084 systems respectively.

We decomposed the MM/GBSA binding free energy into residue-wise contributions, focusing on residues that demonstrated significant binding disruptions greater than 0.5 kcal/mol. Mutated residues were also evaluated for their impact on resistance. Thus, we compared the residue contributions from the secondary mutant relative to the reference G12C mutant using the $\Delta \Delta G^{Res}$values as depicted in Figure 7. Finally, we correlated the residue-wise binding free energy contributions with the residue-wise interaction contacts to identify the key factors contributing to inhibitor resistance across all four KRAS mutants (Figure 8–11).

Our results demonstrated two distinct patterns of resistance: mutated residues caused direct disruptions to binding and indirect effects on surrounding key residues. Although the interactions varied among different inhibitors, the pattern of resistance remained consistent. The mutations in KRAS G12C-Y96D and G12C-Y96S cause direct disruption by replacing the critical Y96 residue, significantly affecting the binding of sotorasib, adagrasib, and MK-1084, resulting in high resistance. In contrast, mutants G12C-G13D and G12C-Q99L exhibited varying levels of resistance (low/moderate and high, respectively) based on the indirect influence of the mutated residues on the binding environment.

### Direct disruption mechanism on KRAS G12C-Y96D and KRAS G12C-Y96S

The direct disruption mechanism pattern was captured in mutant KRAS G12C-Y96D and G12C-Y96S for sotorasib, adagrasib and MK-1084 inhibitor. The Key residues and interactions driving the resistance are presented in Figure 8 and 9 for the mutant G12C-Y96D and G12C-Y96S, respectively.

### Effect of secondary mutant G12C-Y96D

In the sotorasib–G12C-Y96D system, a high level of resistance (ΔΔ*G*=+4.90 kcal/mol) arises primarily from the loss of favorable hydrophobic interactions, which disrupts inhibitor binding. Per-residue energy decomposition revealed that the D96 residue contributes significantly to this reduced affinity (+4.56 kcal/mol). Compared to the reference G12C mutant with Y96, the Y96D mutation results in a dramatic loss of key interactions: –18.24 of hydrophobic contacts, and a loss of –0.53 of π–π stacking. This loss clearly shows that the mutated residue Asp96 directly and significantly influences the inhibitor binding disruption.

In the adagrasib G12C-Y96D system, a significant level of resistance emerges, characterized by a ΔΔG of +3.80 kcal/mol. This resistance stems from a notable reduction in the hydrophobic interactions involving Asp96, Tyr64, and His95, as well as a decrease in the critical hydrogen bonds with Lys16. Unlike sotorasib, adagrasib does not experience a significant decline in π–π stacking interactions with Tyr96, but with Tyr64 showing only a minor change of –0.06. Compared with sotorasib (ΔΔ*G* of +4.90 kcal/mol), adagrasib’s binding approach to KRAS was more spread out, leading to less overall binding disruption. Nonetheless, the contribution of Asp96 remained considerably high at +3.42 kcal/mol, with hydrophobic interactions showing an average decrease of –3.99 contacts per frame. Additionally, the important hydrogen bond with Lys16 also decreases by –0.93 contacts, indicating that both hydrophobic and hydrogen bond interactions play a significant role in binding disruption, thereby contributing to the high resistance of adagrasib.

In the case of MK1084 systems, our findings reveal a significant level of resistance associated with the KRAS G12C-Y96D (ΔΔ*G*=+5.57 kcal/mol) secondary mutants, which is comparable to the resistance observed with sotorasib. The loss of crucial hydrophobic interactions (–13.34) and π–π stacking (–0.11) involving the mutated residues Y96 impairs binding, resulting in resistance to MK-1084. Consequently, we predicted that MK-1084 faces considerable resistance due to the altered hydrophobic interactions stemming from the mutation of Tyr96, which disrupted binding and led to resistance against MK-1084.

### Effect of secondary mutant G12C-Y96S

Similar to the secondary mutant G12C-Y96D, in the Sotorasib KRAS G12C-Y96S system, mutation from Tyr to Ser resulted in high resistance (ΔΔ*G*=+4.62 kcal/mol) due to the loss of hydrophobic interaction and of π–π stacking interaction, primarily contributed from the mutated Ser96 residue. Ser96 contributes highly (+4.33 kcal/mol) because it loses –18.24 and –0.53 average contacts per frame of hydrophobic and π–π stacking interaction respectively. This direct contribution to binding disruption highlights the importance of Tyr96 in maintaining stable binding with sotorasib due to its favorable hydrophobic interactions. As such, a mutation in this residue causes significant disruption, conferring the acquired resistance of sotorasib.

Adagrasib KRAS G12C-Y96S exhibits a significant level of resistance (ΔΔ*G*=+3.17 kcal/mol), which, although lower than the resistance observed in the G12C-Y96D adagrasib system, still indicated notable disruption. The mutated residue Ser96 contributed significantly to this binding disruption with a value of +3.32 kcal/mol, coupled with a reduction of –5.72 in hydrophobic contacts. This binding disruption primarily arose from the diminished hydrophobic contacts involving Ser96, highlighting the importance of this interaction in maintaining ligand stability and preventing resistance.

In the MK1084 systems, our results showed a high resistance level for KRAS G12C-Y96S (ΔΔ*G*=+3.89 kcal/mol) secondary mutants, similar to the observed resistance with sotorasib. The loss of significant hydrophobic (–14.21) and π–π stacking (–0.11) interactions involving the mutated residue S96 disrupted binding, leading to resistance against MK-1084. MK-1084 experienced considerable resistance due to the altered hydrophobic interaction. This underscores the critical role of this residue and its interactions in the development of next-generation inhibitors, highlighting the necessity to consider mutational effects during drug design.

### Indirect disruption mechanism on KRAS G12C-G13D and KRAS G12C-Q99L

The indirect disruption mechanism pattern was captured in the mutant KRAS G12C-Y96D and G12C-Y96S concerning the inhibitors sotorasib, adagrasib, and MK-1084. Key residues and interactions driving the resistance are presented in Figure 10 and 11 for mutant G12C-G13D and G12C-Q99L respectively.

### Effect of secondary mutant G12C-G13D

In G12C-G13D Sotorasib system, the mutation of Gly13 to Asp13 introduced a high electrostatic energy difference disrupting the neighboring binding environment, resulting high resistance level (ΔΔ*G*=+3.02 kcal/mol). The mutated residue Asp13 contributes to the increase of +3.62 kcal/mol electrostatic energy of the protein-ligand complex, indirectly impacting the average loss of –0.49 π-cation contacts of ARG68 residue and –2.56 hydrophobic interaction of Gln99 with sotorasib.

Unlike sotorasib, adagrasib exhibited a moderately low resistance level against the secondary mutant G12C-G13D (ΔΔ*G*=+1.63 kcal/mol), which is below the 2.74 kcal/mol threshold. Adagrasib does not experience a decrease in π-cation interactions from Arg68; instead, it shows an increase of +2.21 hydrophobic contacts, distinguishing it from sotorasib. However, the introduction of the negatively charged Asp13 disrupts the critical hydrogen bonding with Lys16, resulting in a decrease of –0.93 contacts. This reduction contributes to the moderately low resistance level of adagrasib, in contrast to the very low resistance level observed with sotorasib G12C-Q99L (ΔΔG=+0.11 kcal/mol).

For the G12C-G13D MK-1084 systems, we observed a significant resistance with a ΔΔG of +4.03 kcal/mol due to the substitution of Gly13 with Asp13. While the individual contribution of Asp13 resulted in a decrease in resistance of –1.92 kcal/mol, the system ultimately lost the critical hydrogen bond with Lys16, accounting for an increase of +2.89 kcal/mol. This effect counteracted the benefit provided by Asp13. This suggests that Asp13 disrupted the binding environment, as indicated by the electrostatic energy contribution of –14.03 kcal/mol, which interfered with the Lys16 hydrogen bond with MK-1084. Thus, the secondary G13D mutation does not directly hinder binding with MK-1084; instead, it alters the interactions of other residues with the ligand, leading to increased resistance.

### Effect of secondary mutant G12C-Q99L

In contrast to the other three mutants, the KRAS G12C-Q99L sotorasib system exhibited low resistance (ΔΔ*G*=+0.27 kcal/mol) due to an increase in hydrophobic interactions resulting from the substitution of Gln with Leu. This change led to a +9.21 increase in hydrophobic contacts attributable to Leu99. While the mutation reduced the hydrogen bonding of Gln99 with sotorasib, Leu99 offered a more effective hydrophobic surface for the ligand compared to the original polar Gln99.

Adagrasib exhibited a considerable resistance profile, highlighted by a ΔΔG value of +2.96 kcal/mol resulting from the secondary mutation Q99L. This value signifies a marked deviation from the binding characteristics observed for sotorasib. Within the context of KRAS G12C-Q99L, the switch II loop, comprising residues 60–74, displays a notably high root mean square fluctuation (RMSF) value. This elevated RMSF suggests that the switch II loop is considerably more flexible in adagrasib compared to its counterparts, which has meaningful implications for binding dynamics. This increased flexibility manifested itself in various residues that play a pivotal role in reducing binding affinity, particularly through compromised hydrophobic interactions. Specifically, the interactions involving the residues Met72, Glu62, and Tyr64, all integral components of the switch II loop, showed a substantial reduction in hydrophobic contact. Although the substitution of Gln99 with Leu99 did not directly disrupt the binding, it had an indirect yet noteworthy influence on the flexibility of adjacent residues within the switch II loop. Interestingly, while the Leu99 residue resulting from the mutation contributed a minimal binding energy of –0.07 kcal/mol, it did not significantly impair overall binding affinity. Instead, this specific point mutation promotes a higher degree of flexibility in the KRAS protein structure itself. Thus, these indirect effects reinforced the notion that adagrasib was not inherently predisposed to resistance mechanisms associated with the secondary mutation Q99L, illustrating a complex interplay between mutation, protein dynamics, and drug resistance.

MK-1084 exhibited a significant degree of resistance (ΔΔ*G*=+3.86 kcal/mol) due to the secondary mutation KRAS G12C-Q99L. This mutated residue indirectly affected the binding environment, as indicated by its low contribution (–0.16 kcal/mol). However, the system experienced an Arg68 contribution of +3.79 kcal/mol, resulting from a decrease in π-cation average contacts of –0.28. Although the Leu99 substitution enhanced hydrophobic interactions (+9.02), this increase was insufficient to offset the loss of interactions with other residues. This illustrated that the G12C-Q99L mutation in MK-1084 was similar to adagrasib, as evidenced by the high root-mean-square fluctuation (RMSF) value in the SWITCH II loop region. This elevated RMSF indicated that loop flexibility influenced inhibitor binding disruption, as the substitution of Q99 with L99 affected the flexibility of the protein loop, thereby indirectly contributing to resistance by disrupting binding.

### Insight into resistance variations

Several key residues within the KRAS mutant pocket (K16, R68, Y96) serve as critical anchors that maintain ligand stability within the binding pocket (Table 3, Supplementary Figure S1, S2, S4–S13). High-level drug resistance correlates with elevated root-mean-square fluctuation (RMSF) values in the Switch-II loop region, which directly destabilizes the KRAS binding pocket. Direct mutations at these anchor sites, such as the Y96D substitution, markedly increase loop flexibility and subsequent ligand instability. Conversely, indirect resistance mechanisms stem from distal residue substitutions that disrupt interactions with these key anchors. This destabilization loosens the pocket framework and induces severe structural instability, as seen in the G13D and Q99L mutants. The degree of resistance variation (high, moderate, low) depends heavily on how these mutations drive conformational changes that break critical protein-ligand interactions. Because different inhibitors elicit distinct conformational responses, ligand-specific interactions within the pocket dictate the overall resistance profile.

The direct effect of key binding residue Y96 mutated to D/S owing to the resistance of all inhibitors in this study. Key interactions includes hydrophobic (around residue Y96), π–π stacking (Y96) and hydrogen bond (K16). A such, maintaining these interactions in the next drug discovery of anti-resistant inhibitors due to secondary mutation is critical. Although MK-1084 is effective in the single mutant KRAS G12C, secondary mutation in Y96 impairs the critical π–π stacking and surrounding neighboring residues (hydrophobic) leading to resistance.

The indirect effect of residue G13 and Q99L caused variations in the resistance level. Although our protein-ligand analysis have limitations in capturing residue to residue interaction between mutated residue and other key residues, we have shown the changes that occurs in the other key residues with the inhibitors. The replacement of G13 to D13 and Q99 with L99 caused different effect by indirect disruption. Changes in electrostatic energy in the p-loop regions (G13) incurs flexibility in the switch regions, causing the KRAS switch flexibility which further impairs the binding with inhibitor owing high resistance to sotorasib and MK-1084 and moderate resistance to adagrasib.

The Q99L mutation induces low resistance against sotorasib because it leaves the core K16, R68, and Y96 interaction network undisturbed (Figure 11, Supplementary Figure S11). In contrast, the Q99L mutation confers high resistance to adagrasib by selectively disrupting the K16 hydrogen bond, causing the ligand to deviate from its optimal binding posture due to this lost interaction (Figure 11, Supplementary Figure S12). These phenotypic differences arise from the distinct binding conformations of the sotorasib and adagrasib system [40]. Lys16 (K16) is a highly specialized anchor situated directly at the nucleotide-binding site near the P-loop and the bound GDP/GTP molecule. Sotorasib’s core architecture enables tight interaction with the P-loop region, specifically forming a critical, direct hydrogen bond between its ketonic carbonyl group and the side chain of Lys16. Adagrasib adopts a more extended conformation directed toward the Switch-II loop, relying on an indirect, potentially water-mediated alignment near Lys16. Because adagrasib’s primary anchors are located elsewhere (such as the Q99 network), remote mutations that induce allosteric conformational changes exert a tugging force on the drug, causing its weaker interaction with Lys16 to snap entirely. Based on this insight, the development of next anti-resistant drug is suggested to consider these key anchors and its interaction.

## Conclusion

In this study, we conducted a systematic examination of how secondary mutations influence the resistance profiles of KRAS G12C inhibitors, specifically Sotorasib, Adagrasib, and the next-generation inhibitor MK-1084. Molecular dynamics simulations and binding free energy analyses indicated varying degrees of resistance associated with each mutant. All three inhibitors exhibited a high degree of resistance due to the secondary mutations G12C-Y96D and G12C-Y96S. Sotorasib proved effective against the secondary mutant KRAS G12C-Q99L, while adagrasib showed efficacy against the secondary mutant KRAS G12C-G13D, as evidenced by the moderately low values of relative binding free energy, indicating reduced resistance levels. In contrast, MK-1084 displayed a high level of acquired resistance against all four secondary mutants (G12C-Y96D, G12C-Y96S, G12C-G13D, and G12C-Q99L), as reflected by its relatively high relative binding free energy values. Although MK-1084 is effective in inhibiting KRAS G12C, it faces significant challenges due to acquired resistance from secondary mutations.

The computational predictions for sotorasib and adagrasib closely matched the experimental data, confirming the validity of the proposed approach. The strong correlation and low error metrics highlighted the reliability of this method for evaluating resistance. Furthermore, predictions for MK-1084 indicated its reduced efficacy against resistant variants, providing valuable insights for the future development of KRAS inhibitors.

Per-residue decomposition and interaction analyses provided molecular-level insights into the altered binding landscapes contributing to resistance. We identified two major patterns in the mechanism of resistance involving the direct and indirect contribution of the mutated residue to the disruption of the inhibitor binding. Alterations at specific residues interfered with inhibitor binding, causing both direct disruption and indirect effects on neighboring critical residues. Although the exact interactions differed between inhibitors, the overall resistance trends remained consistent. The secondary mutant KRAS G12C-Y96D and G12C-Y96S directly impaired binding by substituting the essential Y96 residue, leading to significant resistance to sotorasib, adagrasib, and MK-1084. In contrast, G12C-G13D and G12C-Q99L variants induced varying resistance levels through indirect structural changes in the KRAS binding pocket. These findings underscore the importance of incorporating dynamic structural analyses into drug resistance research. This approach is crucial for guiding the rational design of more resilient KRAS-targeted therapies capable of effectively addressing the complexities associated with resistance mechanisms.

## Conflict of interest

Riksa Meidy Karim, Kazutomo Kawaguchi, Hidemi Nagao declare that they have no conflict of interest.

## Author contributions

Riksa Meidy Karim, Kazutomo Kawaguchi, Hidemi Nagao directed the entire project and co-wrote the manuscript.

## Data availability

The evidence data generated and/or analyzed during the current study are available from the corresponding author on reasonable request.

## Figures and Tables

**Table 1:** Backbone RMSD values obtained from structural superimposition of KRAS secondary mutant complexes onto the reference KRAS G12C crystal structure prior to the MD simulations

KRAS Mutant	Sotorasib System	Adagrasib System	MK-1084 System
G12C	—	—	—
G12C-Y96D	0.427	0.396	0.450
G12C-Y96S	0.398	0.402	0.385
G12C-G13D	0.417	0.359	0.437
G12C-Q99L	0.430	0.424	0.388

PDB reference structures (Sotorasib: 6OIM, Adagrasib: 6UT0, MK-1084: 8S8C)

**Table 2:** Experimental and MM/GBSA binding free energy values

Inhibitor	KRAS Mutant	Δ*G*	Δ*G*_exp_ [5]	ΔΔ*G*	ΔΔ*G*_exp_
Sotorasib	**G12C**	–76.55	–10.85	—	—
	**G12C-Y96D**	–71.65	–6.86	4.90	3.99
	**G12C-Y96S**	–71.93	–6.86	4.62	3.99
	**G12C-G13D**	–73.52	–7.60	3.02	3.26
	**G12C-Q99L**	–76.27	–9.98	0.27	0.87
Adagrasib	**G12C**	–83.22	–12.20	—	—
	**G12C-Y96D**	–79.43	–8.49	3.80	3.71
	**G12C-Y96S**	–80.05	–8.73	3.17	3.46
	**G12C-G13D**	–81.59	–10.38	1.63	1.81
	**G12C-Q99L**	–80.27	–8.78	2.96	3.41
MK-1084	**G12C**	–85.35	—	—	—
	**G12C-Y96D**	–79.78	—	5.57	—
	**G12C-Y96S**	–81.46	—	3.89	—
	**G12C-G13D**	–81.32	—	4.03	—
	**G12C-Q99L**	–81.49	—	3.86	—

The reported MM/GBSA values represent the averaged results obtained from three independent MD simulations (n=3), with detailed results provided in Supplementary Table S1.

**Table 3:** Resistance Profile of KRAS G12C Secondary Mutation to Inhibitors

Inhibitor	KRAS Secondary Mutation	Resistance Level	Resistance Mechanism	Mutated Residue	Disrupted Anchor Residues	Interaction Loss
Sotorasib	**Y96D**	High	Direct	Y96 to D96	D96	Hydrophobic, π–π stack
	**Y96S**	High	Direct	Y96 to S96	S96	Hydrophobic, π–π stack
	**G13D**	High	Indirect	G13 to D13	R68	π-cation
	**Q99L**	Low	—	Q99 to L99	—	—
Adagrasib	**Y96D**	High	Direct	Y96 to D96	D96	Hydrophobic, π–π stack
	**Y96S**	High	Direct	Y96 to S96	S96	Hydrophobic, π–π stack
	**G13D**	Moderate	Indirect	G13 to D13	K16	Hydrogen Bond
	**Q99L**	High	Indirect	Q99 to L99	K16	Hydrogen Bond
MK-1084	**Y96D**	High	Direct	Y96 to D96	D96	Hydrophobic, π–π stack
	**Y96S**	High	Direct	Y96 to S96	S96	Hydrophobic, π–π stack
	**G13D**	High	Indirect	G13 to D13	K16	Hydrogen Bond
	**Q99L**	High	Indirect	Q99 to L99	R68	π-cation
