# Supplementary material for: Theoretical study of KRAS G12C secondary mutations influencing inhibitor resistance
Source: Biophys Physicobiol. 2026 Jul 9;23(3):e230021. doi: 10.2142/biophysico.bppb-v23.0021 (PMC13542558; doi:10.2142/biophysico.bppb-v23.0021)
Supplement: Supplementary file 1 [file 23_e230021_1.pdf]

Biophysics and Physicobiology
Vol. 23, e230021 (2026)
Supplementary Materials

Supplementary Materials - Theoretical Study of KRAS G12C Secondary Mutations Influencing Inhibitor Resistance

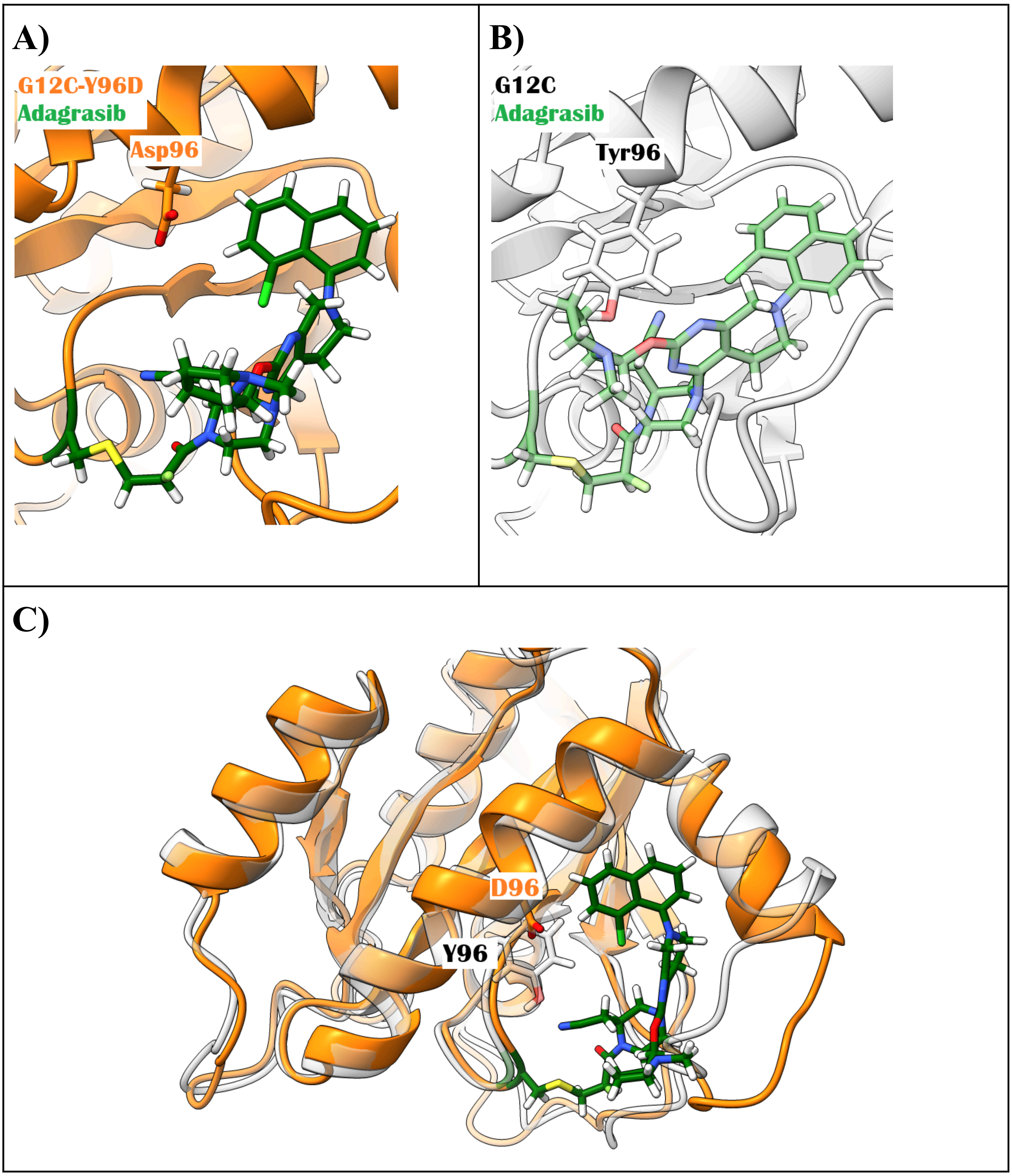

Supplementary Figure S1 Structural impact of the Y96D secondary mutation on adagrasib binding. **(A)** Binding mode visualization of adagrasib (dark green) in the KRAS G12C-Y96D double mutant compared to **(B)** the reference KRAS G12C single mutant. **(C)** Structural overlay of adagrasib-bound KRAS G12C-Y96D (orange) against the KRAS G12C reference complex (grey), highlighting the substitution of the Tyr96 residue (grey) with the smaller, negatively charged mutant Asp96 residue (orange).

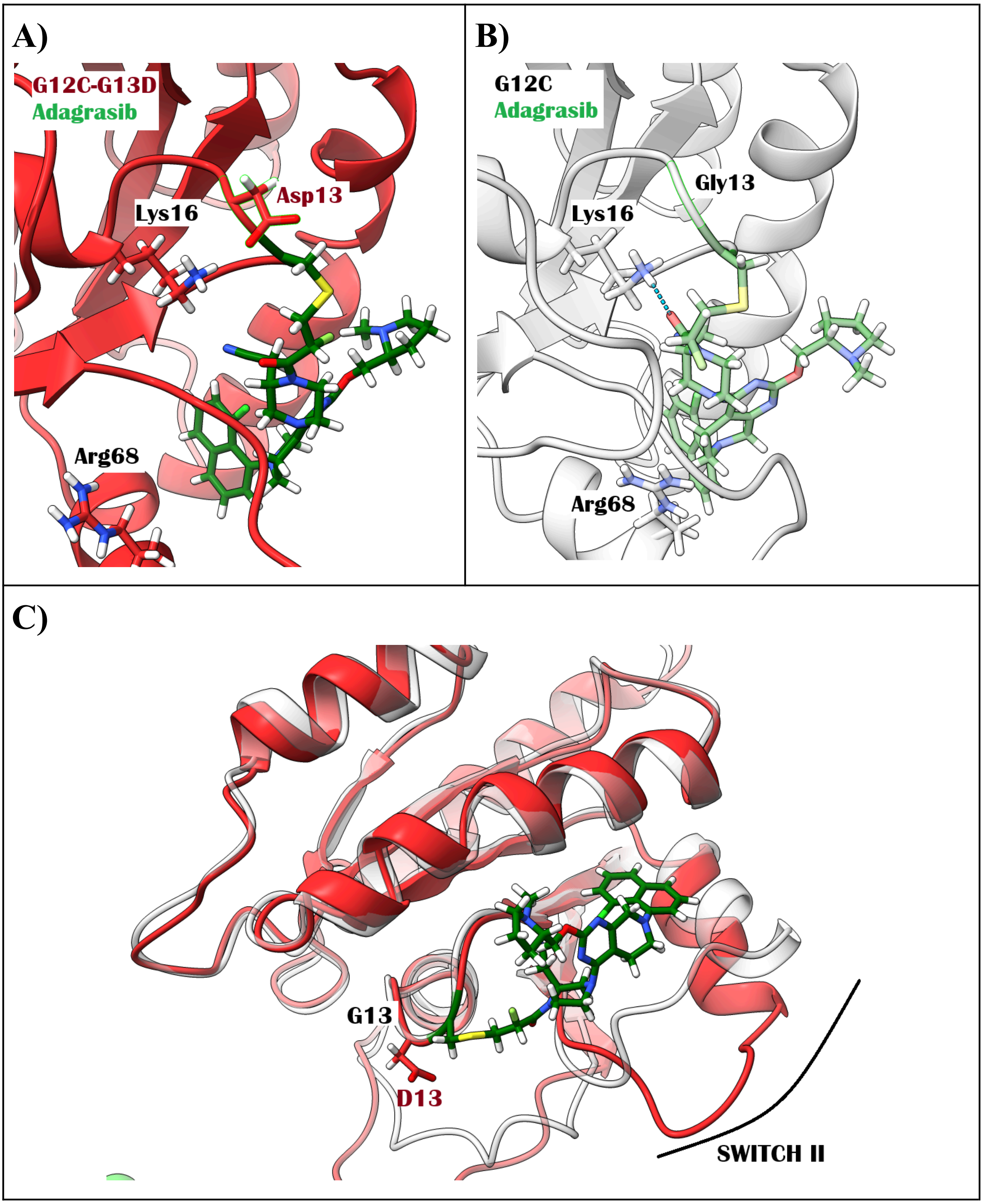

Supplementary Figure S2 Structural impact of the G13D secondary mutation on adagrasib binding. **(A)** Binding mode visualization of adagrasib (dark green) in the KRAS G12C-G13D double mutant compared to **(B)** the reference KRAS G12C single mutant. **(C)** Structural overlay of adagrasib-bound KRAS G12C-G13D (light red) against the KRAS G12C reference complex (grey), highlighting the substitution of the Gly13 residue (grey) with the bulky, charged Asp13 residue (light red) indirectly disrupts the SWITCH II loop conformation via the loss of Lys16 hydrogen bond interaction

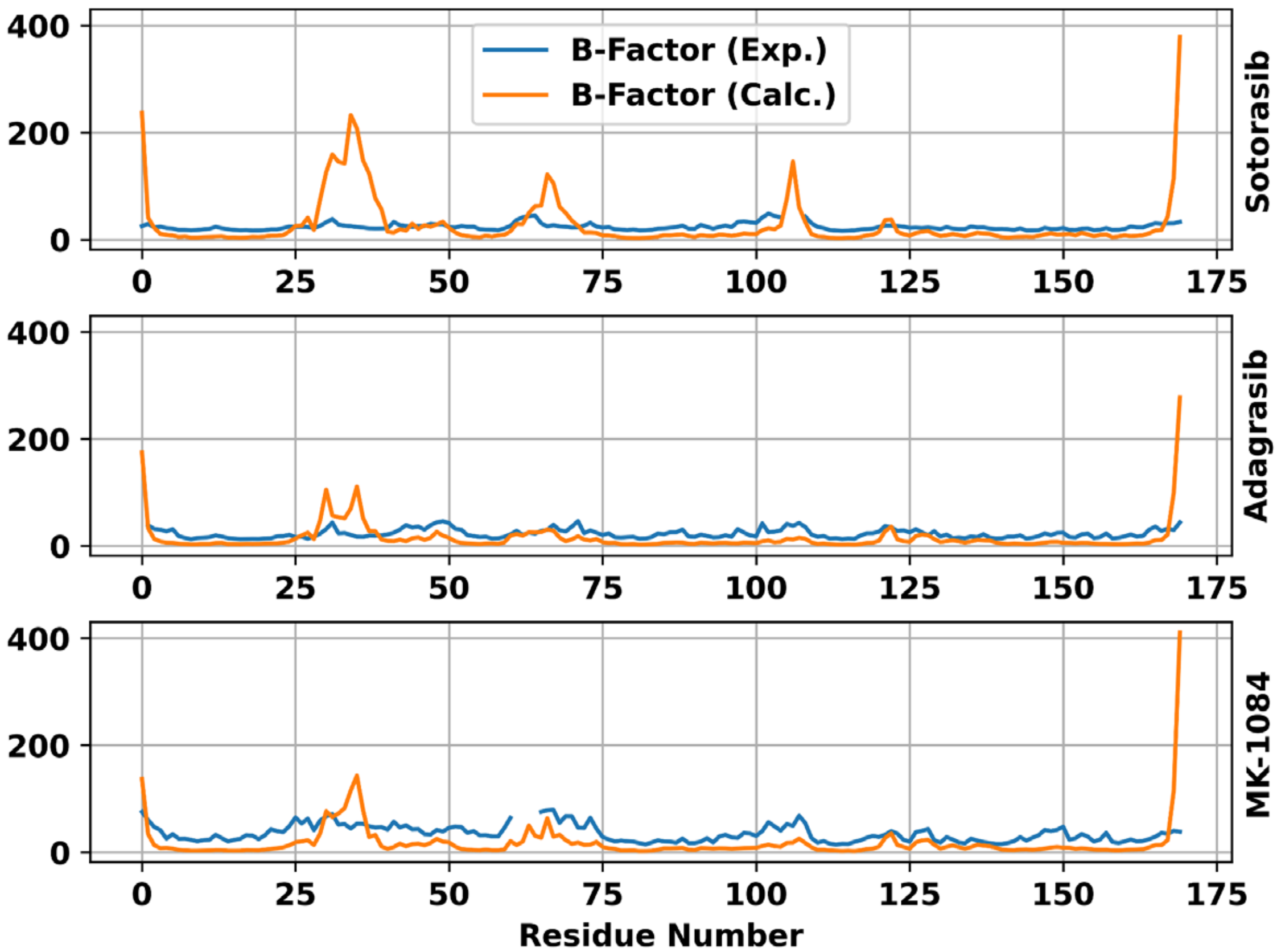

Supplementary Figure S3 Experimental and Calculated B-Factor of KRAS G12C single mutant.

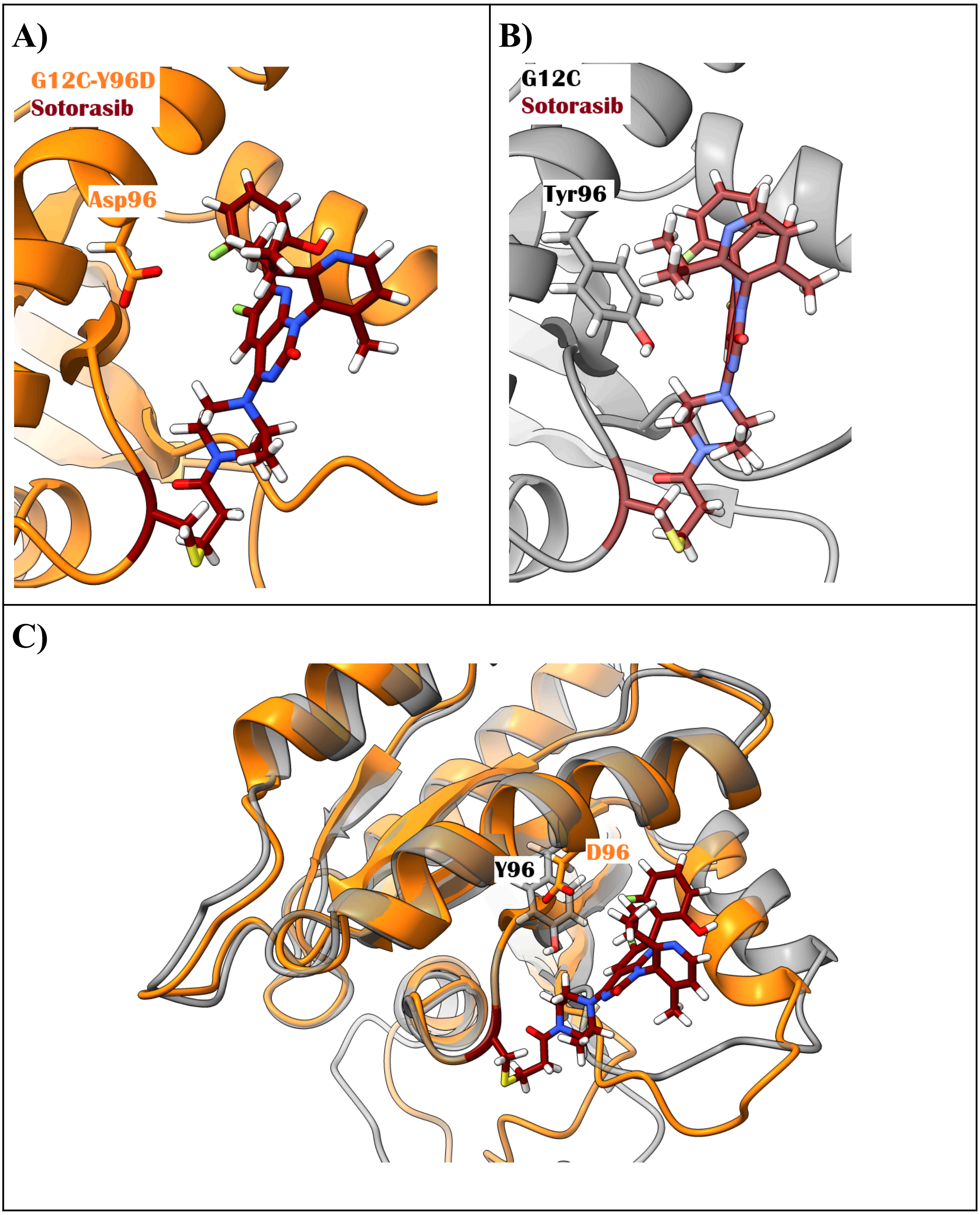

Supplementary Figure S4. Structural impact of the Y96D secondary mutation on sotorasib binding. **(A)** Binding mode visualization of sotorasib (dark red) in the KRAS G12C-Y96D double mutant compared to **(B)** the reference KRAS G12C single mutant. **(C)** Structural overlay of sotorasib-bound KRAS G12C-Y96D (orange) against the KRAS G12C reference complex (grey), highlighting the substitution of the Tyr96 residue (grey) with the smaller, negatively charged mutant Asp96 residue (orange).

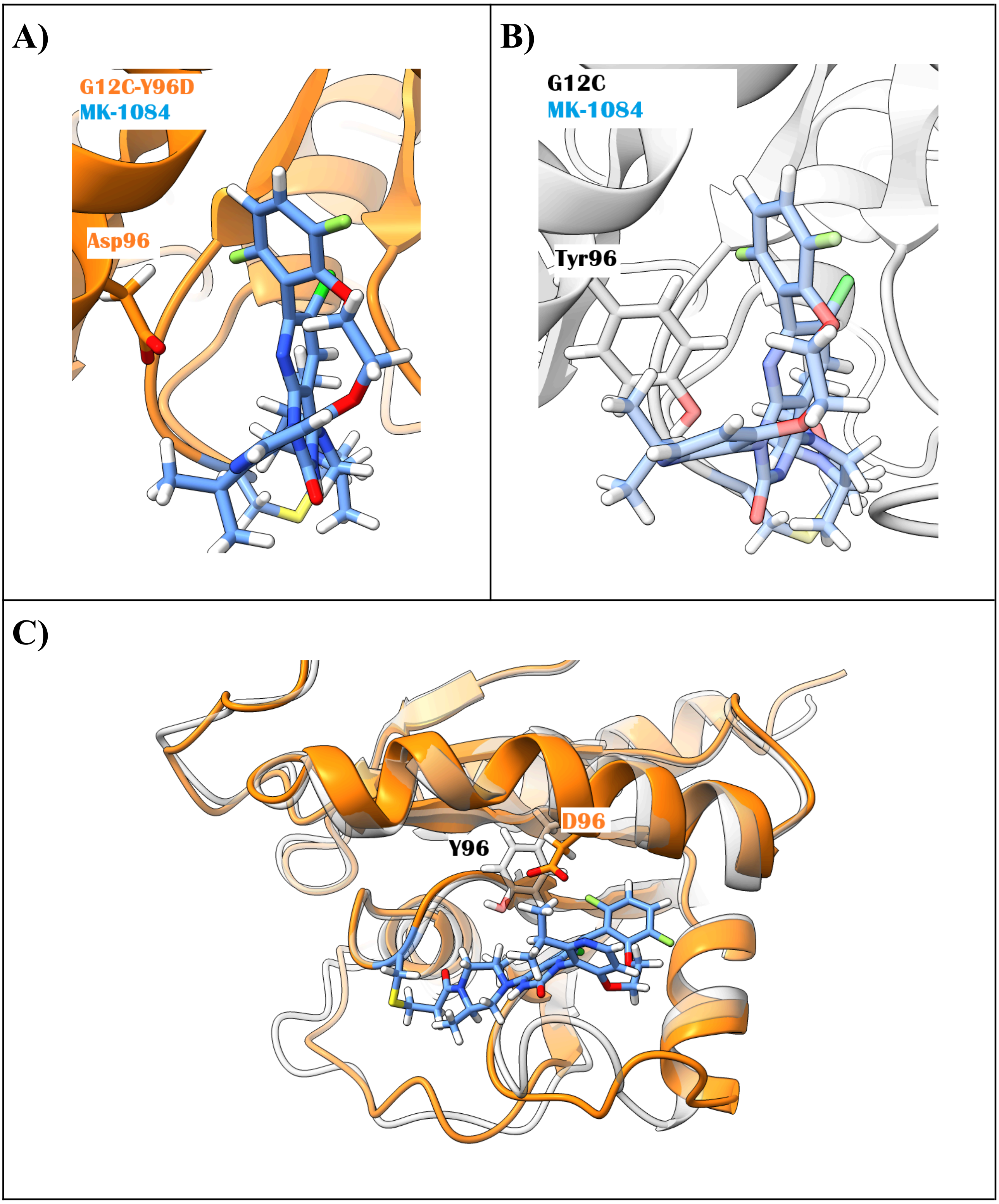

Supplementary Figure S5 Structural impact of the Y96D secondary mutation on MK-1084 binding. **(A)** Binding mode visualization of MK-1084 (blue) in the KRAS G12C-Y96D double mutant compared to **(B)** the reference KRAS G12C single mutant. **(C)** Structural overlay of MK-1084-bound KRAS G12C-Y96D (orange) against the KRAS G12C reference complex (grey), highlighting the substitution of the Tyr96 residue (grey) with the smaller, negatively charged mutant Asp96 residue (orange).

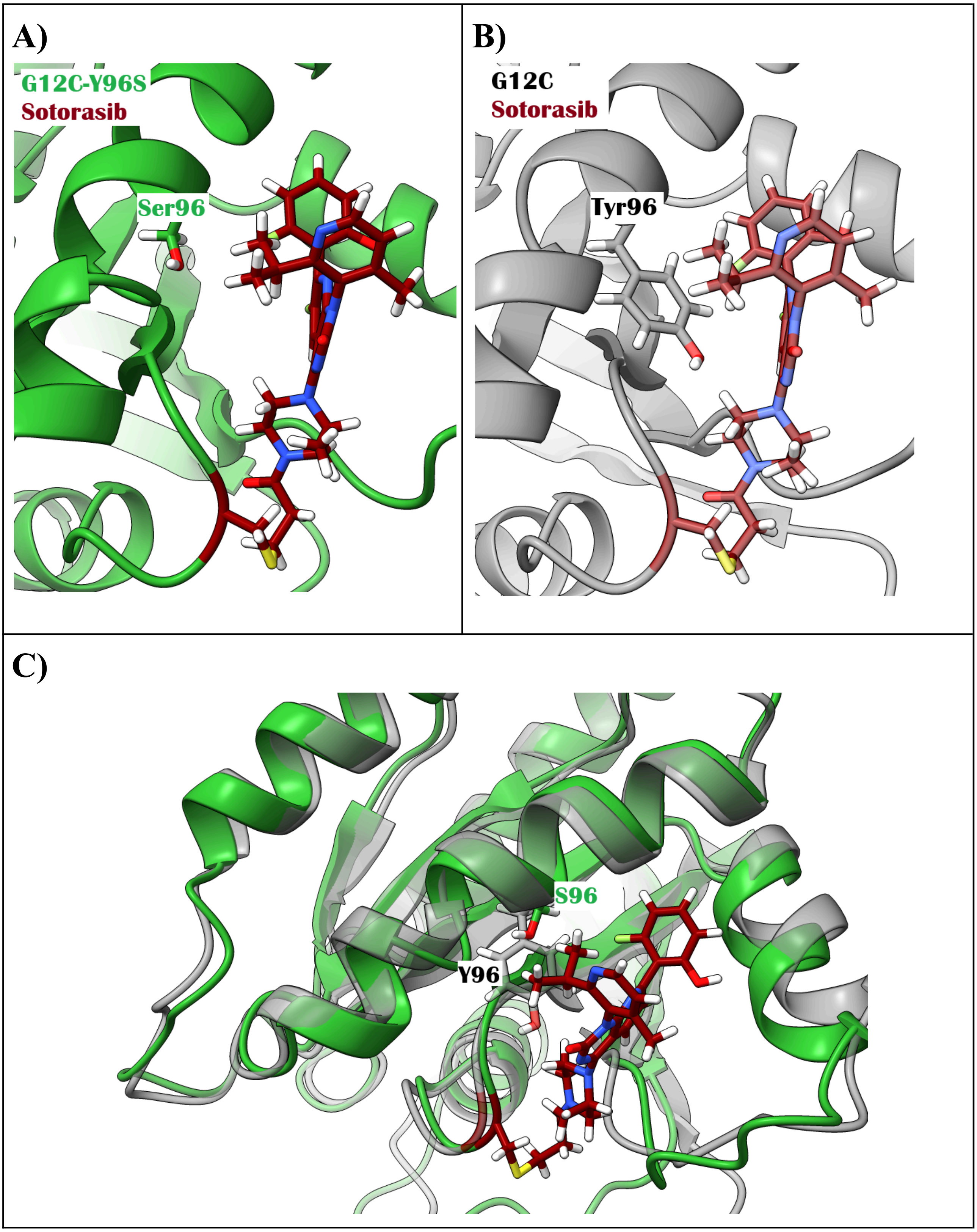

Supplementary Figure S6 Structural impact of the Y96S secondary mutation on sotorasib binding. **(A)** Binding mode visualization of sotorasib (dark red) in the KRAS G12C-Y96S double mutant compared to **(B)** the reference KRAS G12C single mutant. **(C)** Structural overlay of sotorasib-bound KRAS G12C-Y96S (green) against the KRAS G12C reference complex (grey), highlighting the substitution of the Tyr96 residue (grey) with highly truncated Serine 96 mutant residue (green).

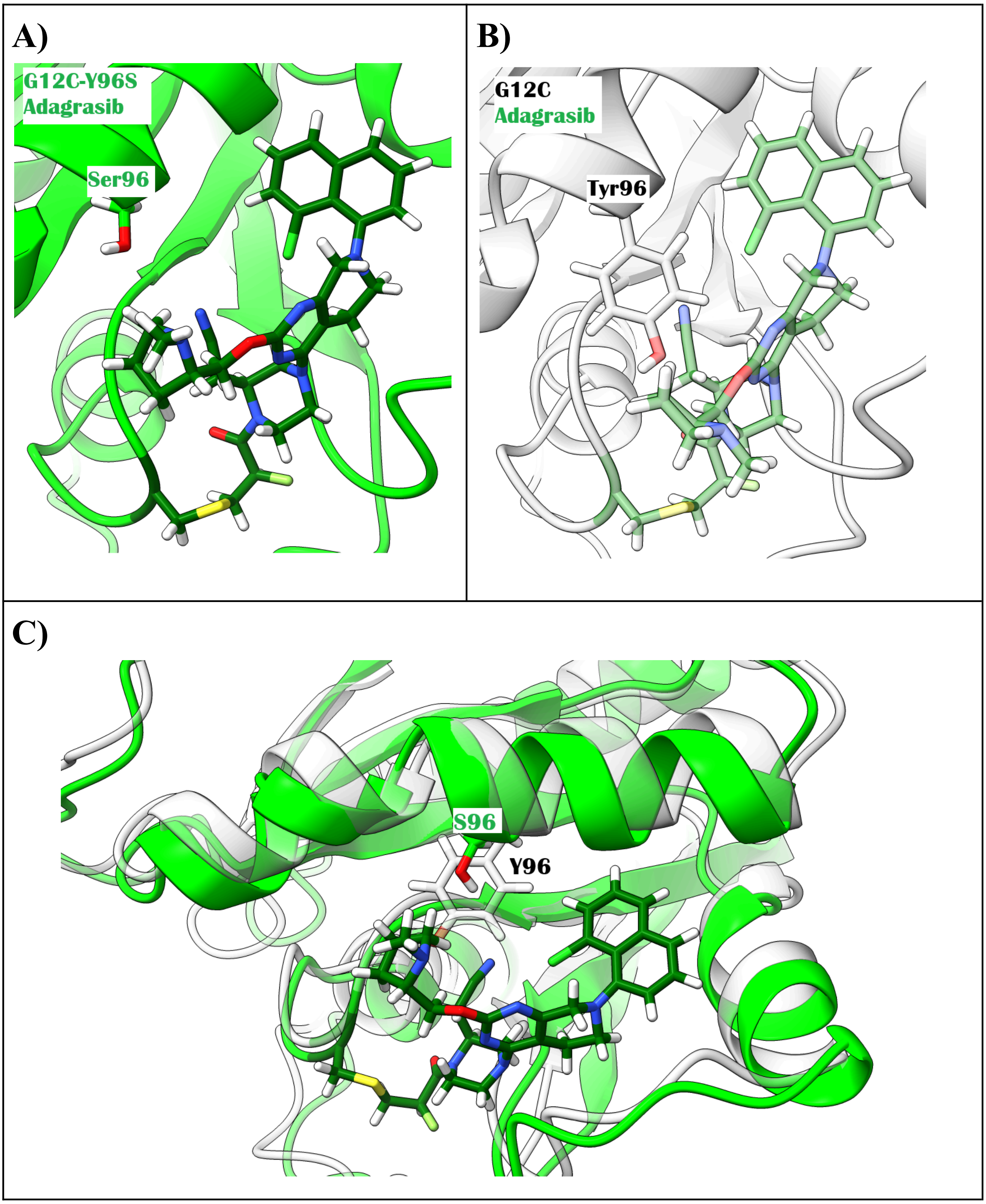

Supplementary Figure S7 Structural impact of the Y96S secondary mutation on adagrasib binding. **(A)** Binding mode visualization of adagrasib (dark green) in the KRAS G12C-Y96S double mutant compared to **(B)** the reference KRAS G12C single mutant. **(C)** Structural overlay of adagrasib-bound KRAS G12C-Y96S (light green) against the KRAS G12C reference complex (grey), highlighting the substitution of the Tyr96 residue (grey) with highly truncated Serine 96 mutant residue (light green).

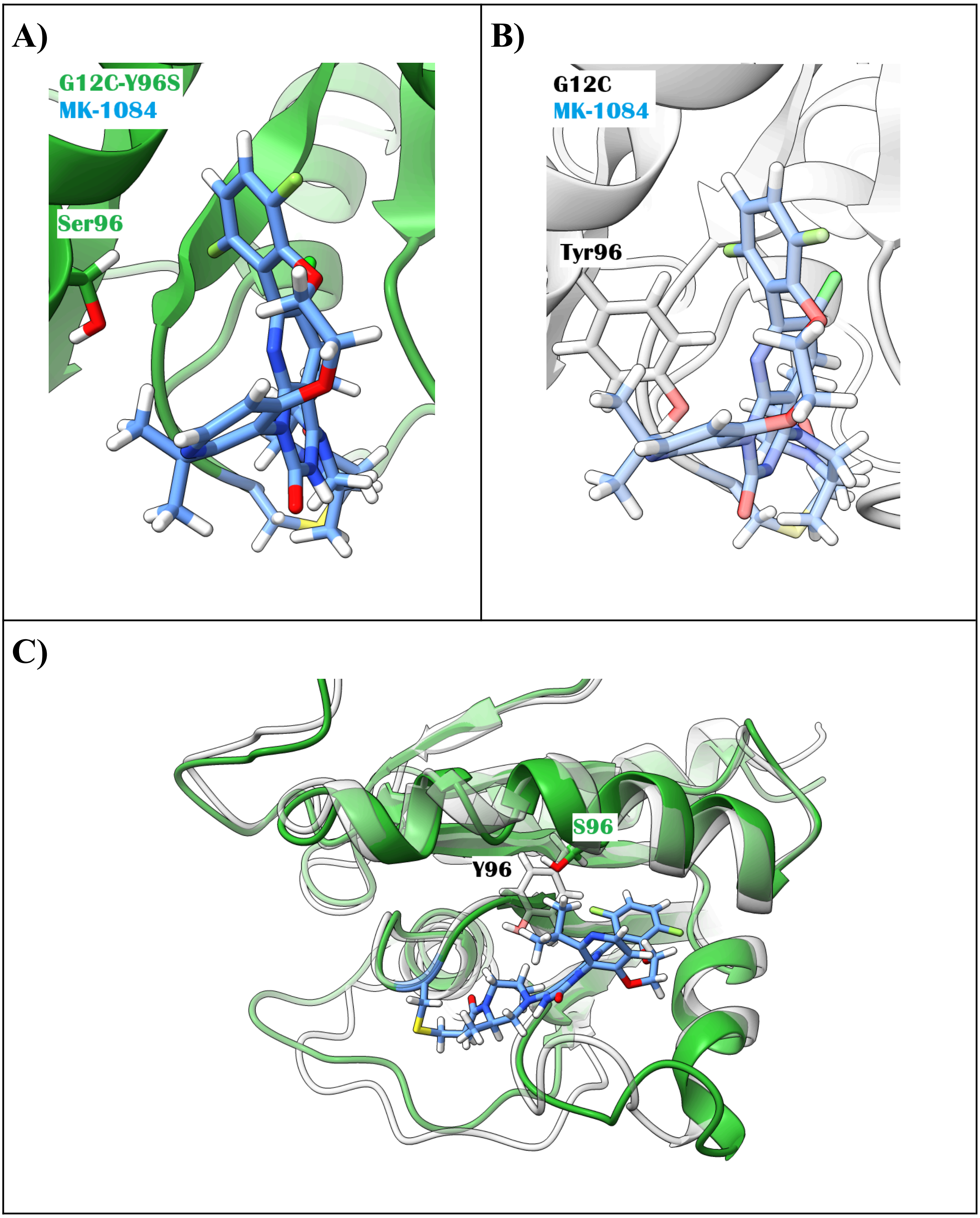

Supplementary Figure S8 Structural impact of the Y96S secondary mutation on MK-1084 binding. **(A)** Binding mode visualization of MK-1084 (blue) in the KRAS G12C-Y96S double mutant compared to **(B)** the reference KRAS G12C single mutant. **(C)** Structural overlay of MK-1084-bound KRAS G12C-Y96S (light green) against the KRAS G12C reference complex (grey), highlighting the substitution of the Tyr96 residue (grey) with highly truncated Serine 96 mutant residue (light green).

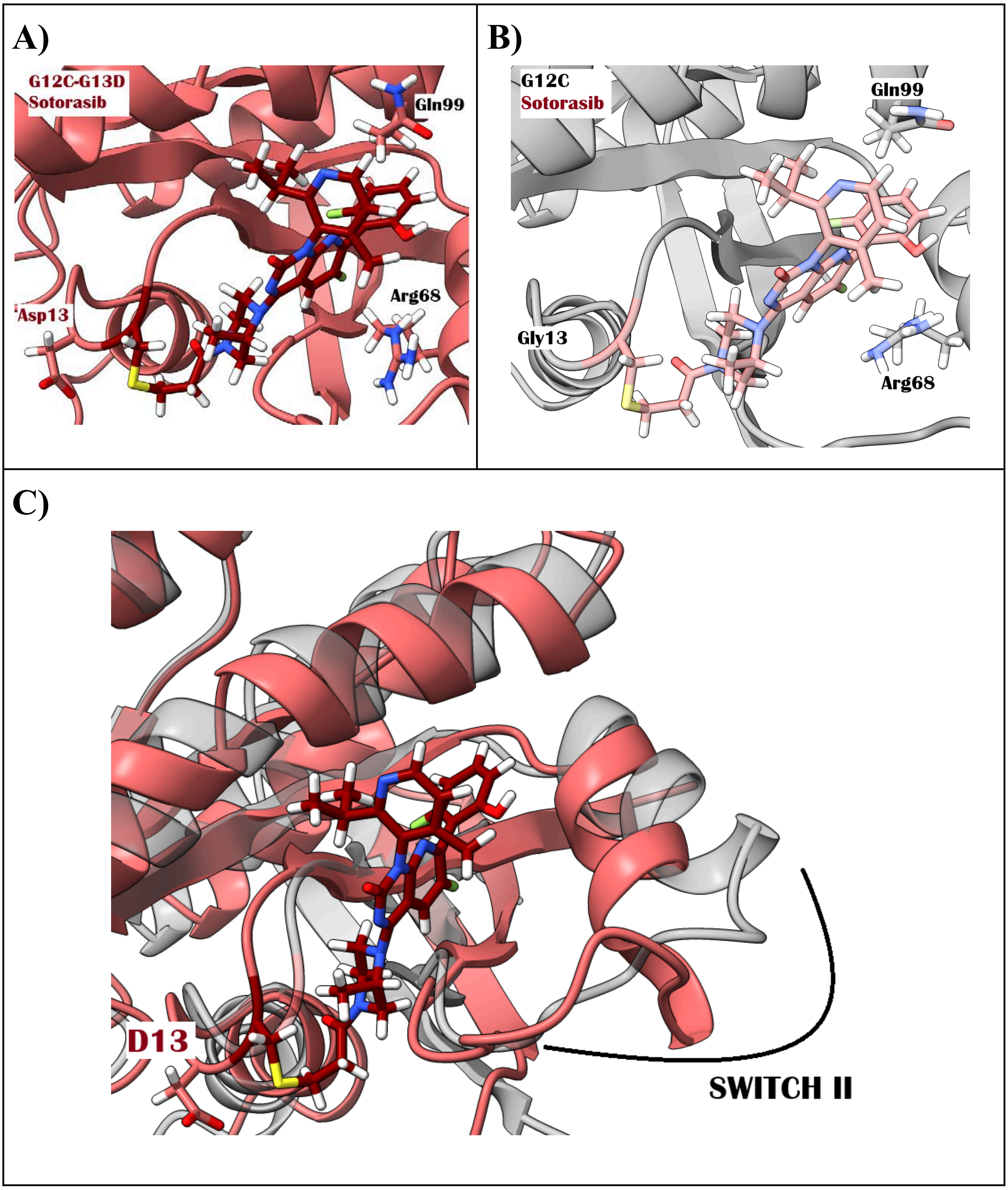

Supplementary Figure S9 Structural impact of the G13D secondary mutation on sotorasib binding. **(A)** Binding mode visualization of sotorasib (dark red) in the KRAS G12C-G13D double mutant compared to **(B)** the reference KRAS G12C single mutant. **(C)** Structural overlay of sotorasib-bound KRAS G12C-G13D (light red) against the KRAS G12C reference complex (grey), highlighting the substitution of the Gly13 residue (grey) with the bulky, charged Asp13 residue (light red) indirectly disrupts the SWITCH II loop conformation via the loss of Arg68 interaction.

.

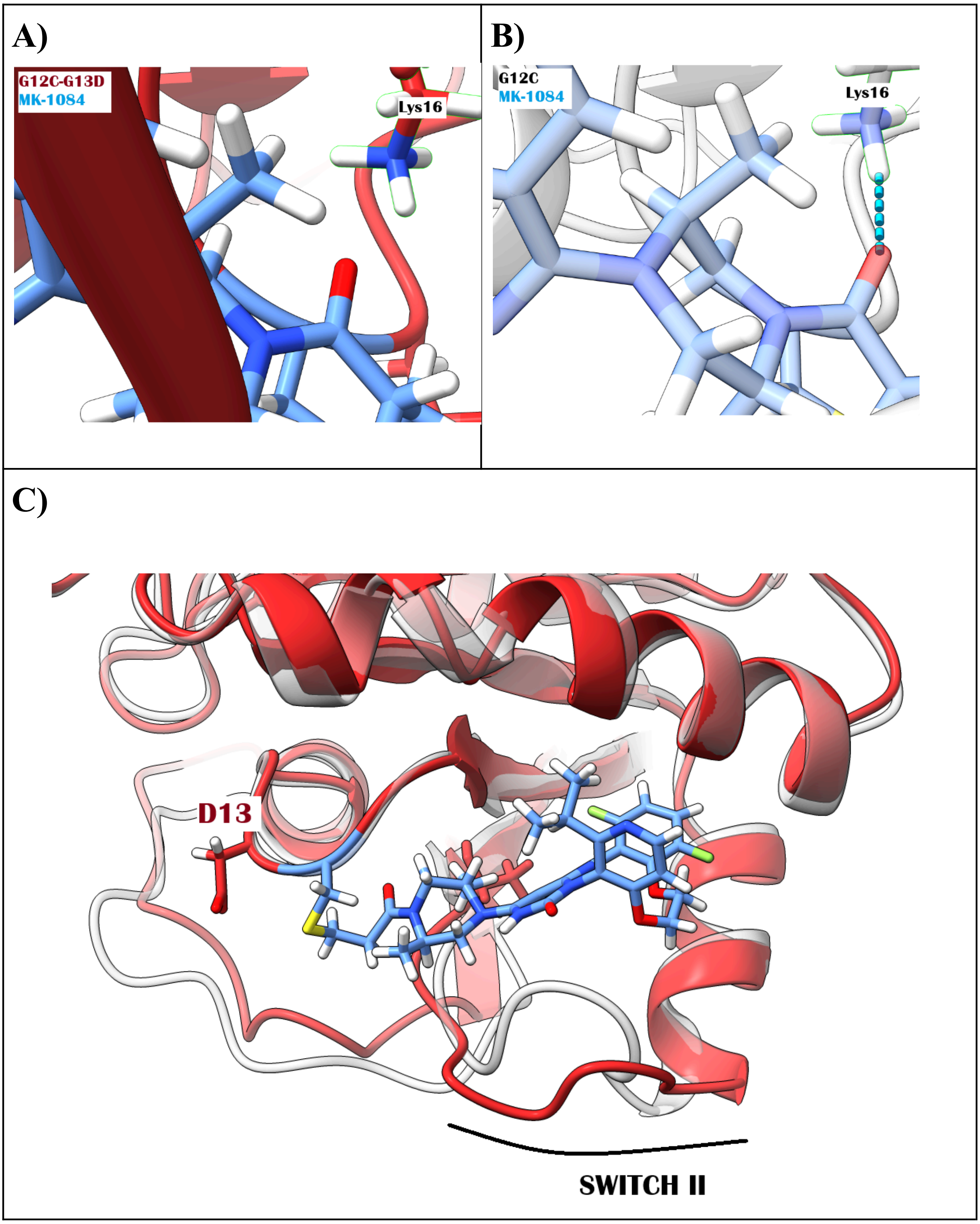

Supplementary Figure S10 Structural impact of the G13D secondary mutation on MK-1084 binding. **(A)** Binding mode visualization of MK-1084 (blue) in the KRAS G12C-G13D double mutant compared to **(B)** the reference KRAS G12C single mutant. **(C)** Structural overlay of MK-1084-bound KRAS G12C-G13D (light red) against the KRAS G12C reference complex (grey), highlighting the substitution of the Gly13 residue (grey) with the bulky, charged Asp13 residue (light red) indirectly disrupts the SWITCH II loop conformation via the loss of Lys16 hydrogen bond interaction.

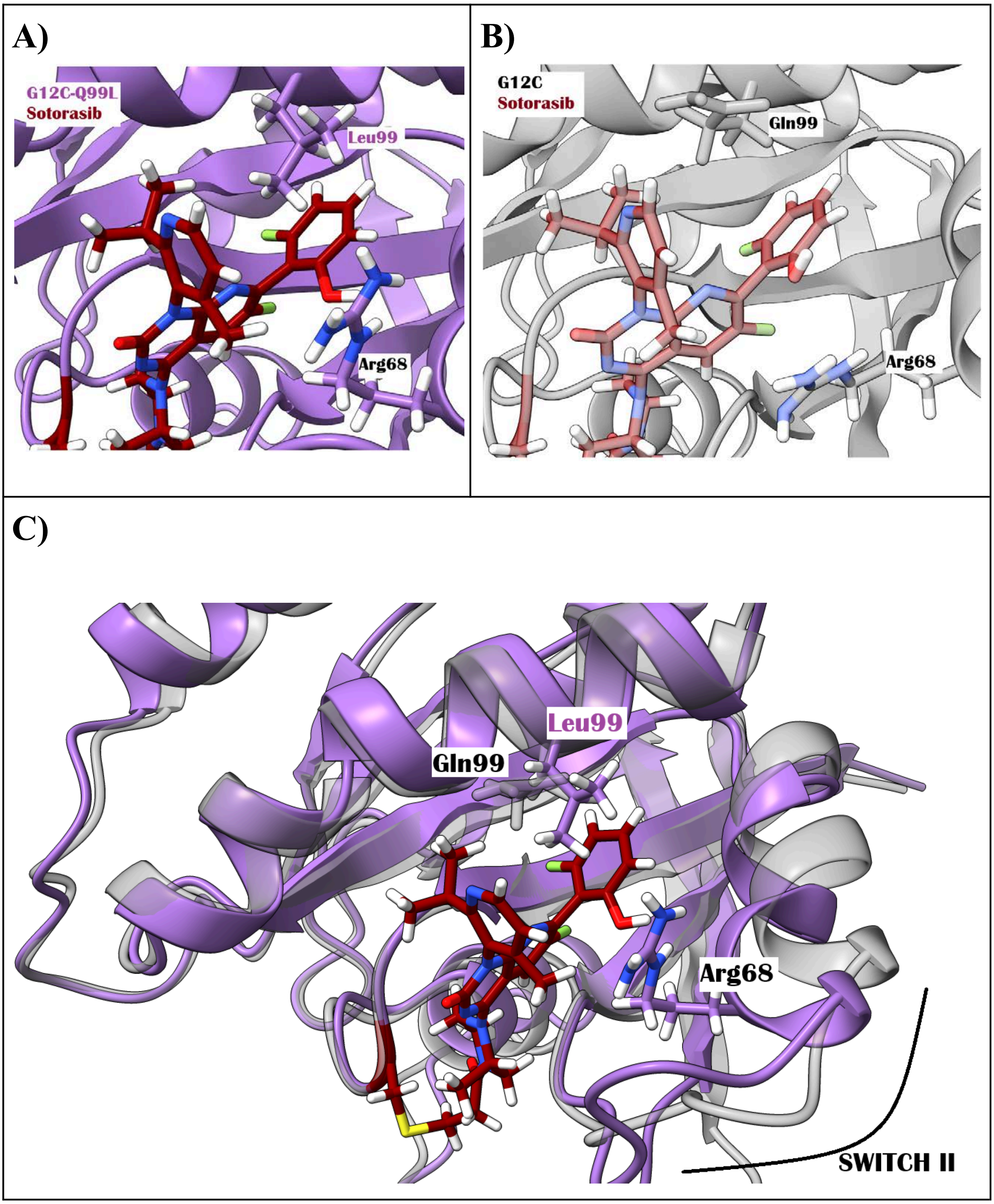

Supplementary Figure S11 Structural impact of the Q99L secondary mutation on sotorasib binding. **(A)** Binding mode visualization of sotorasib (dark red) in the KRAS G12C-Q99L double mutant compared to **(B)** the reference KRAS G12C single mutant. **(C)** Structural overlay of sotorasib-bound KRAS G12C-Q99L (purple) against the KRAS G12C reference complex (grey), highlighting the substitution of the native, polar Gln99 residue (grey) with the hydrophobic Leu99 mutant residue (purple). In contrast to other Switch-II pocket inhibitors that suffer steric or electrostatic disruption from this change, sotorasib maintains a compatible binding mode and steric fit, retaining potent covalent engagement with Cys12 and remaining low resistance to the Q99L.

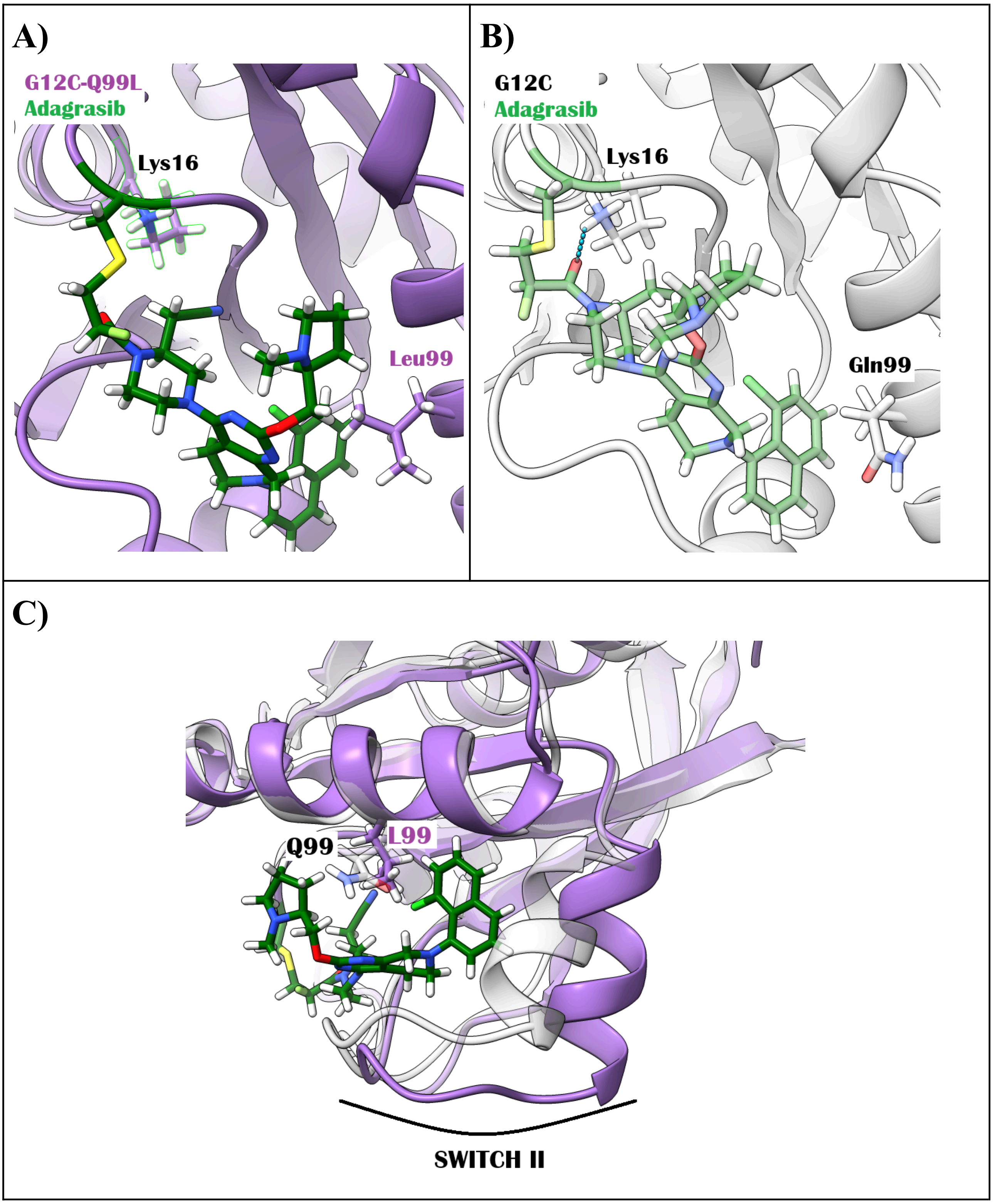

Supplementary Figure S12 Structural impact of the Q99L secondary mutation on adagrasib binding. **(A)** Binding mode visualization of adagrasib (dark green) in the KRAS G12C-Q99L double mutant compared to **(B)** the reference KRAS G12C single mutant. **(C)** Structural overlay of adagrasib-bound KRAS G12C-Q99L (purple) against the KRAS G12C reference complex (grey), highlighting the substitution of the native, polar Gln99 residue (grey) with the hydrophobic Leu99 mutant residue (purple) indirectly disrupts the SWITCH II loop conformation via the loss of Lys16 hydrogen bond interaction.

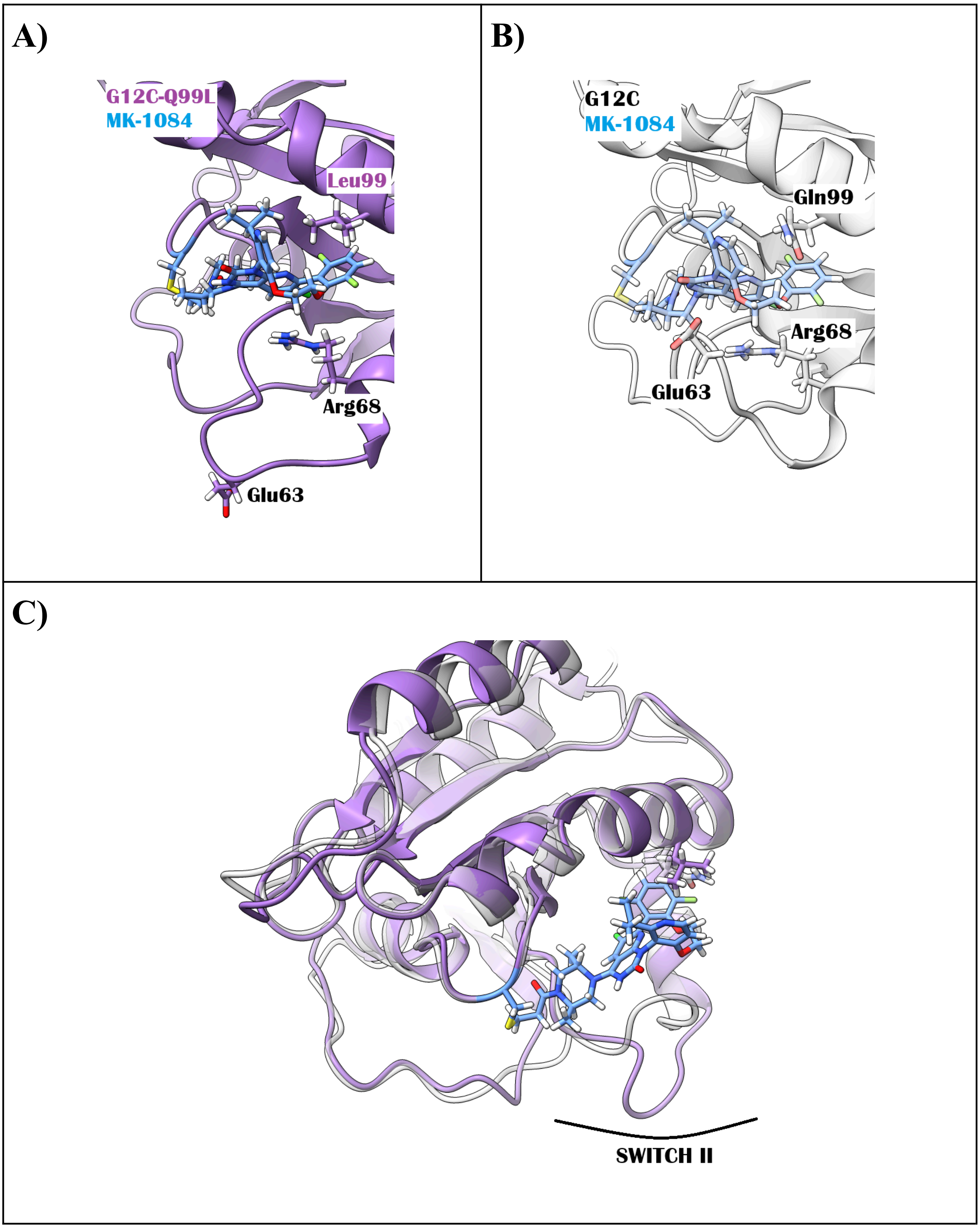

Supplementary Figure S13 Structural impact of the Q99L secondary mutation on MK-1084 binding. **(A)** Binding mode visualization of MK-1084 (blue) in the KRAS G12C-Q99L double mutant compared to **(B)** the reference KRAS G12C single mutant. **(C)** Structural overlay of MK-1084-bound KRAS G12C-Q99L (purple) against the KRAS G12C reference complex (grey), highlighting the substitution of the native, polar Gln99 residue (grey) with the hydrophobic Leu99 mutant residue (purple) indirectly disrupts the SWITCH II loop conformation via the loss of Arg68 and Glu63 interaction

Supplementary Table S1 KRAS Mutant - Inhibitor Binding Free Energies (kcal/mol).

INHIBITOR	MUTANT	ΔG_{exp}	ΔG (N1)	ΔG (N2)	ΔG (N3)	ΔG (Avg.)	ΔG (Std.)	$\Delta\Delta G$	$\Delta\Delta G$ (std.)	$\Delta\Delta G_{exp}$
SOTORASIB	G12C	-10.85	-76.46	-76.52	-76.66	-76.55	0.10	-	-	-
	G12C-Y96D	-6.86	-71.81	-71.16	-71.98	-71.65	0.43	4.90	0.44	3.99
	G12C-Y96S	-6.86	-72.44	-72.29	-71.06	-71.93	0.76	4.62	0.76	3.26
	G12C-G13D	-7.6	-73.54	-72.76	-74.27	-73.52	0.76	3.02	0.76	0.87
	G12C-Q99L	-9.98	-76.35	-76.79	-75.68	-76.27	0.56	0.27	0.57	3.99
ADAGRASIB	G12C	-12.20	-83.39	-82.96	-83.32	-83.22	0.23	-	-	-
	G12C-Y96D	-8.49	-79.42	-80.13	-78.73	-79.43	0.70	3.80	0.74	3.71
	G12C-Y96S	-8.73	-80.45	-80.42	-79.28	-80.05	0.67	3.17	0.71	3.46
	G12C-G13D	-10.38	-81.69	-81.87	-81.21	-81.59	0.34	1.63	0.41	1.81
	G12C-Q99L	-8.78	-80.64	-79.45	-80.71	-80.27	0.71	2.96	0.74	3.41
MK-1084	G12C	-	-85.54	-85.78	-84.73	-85.35	0.55	-	-	-
	G12C-Y96D	-	-79.74	-80.25	-79.34	-79.78	0.46	5.57	0.71	-
	G12C-Y96S	-	-81.33	-80.58	-82.47	-81.46	0.95	3.89	1.10	-
	G12C-G13D	-	-81.8	-81.11	-81.05	-81.32	0.42	4.03	0.69	-
	G12C-Q99L	-	-81.66	-81.2	-81.61	-81.49	0.25	3.86	0.61	-

Supplementary Table S2 Key residue energy decomposition and delta interaction frequency for KRAS G12C-Y96D secondary mutant complex.

Mutant	Residue	Per Residue Decomposed Binding Free energy (kcal / mol)					Delta Interaction Frequency (Contact per Frame)				
		vdW	Ele.	Polar Solv.	Non-polar Solv.	Total	hydrophobic	π–π stack	H-Bond Donor	H-Bond Acceptor	π-cation
G12C-Y96D-SOTORASIB	Asp96	2.25	0.48	-0.13	1.95	4.56	-18.24	-0.53	-	-	-
	Gln61	1.16	0.25	-0.20	1.05	2.26	-0.03	-	-	0.00	-
	Gln99	1.01	0.06	0.02	0.88	1.97	-3.73	-	0.00	-0.12	-
	Glu63	0.54	0.38	-0.32	0.53	1.13	-0.48	-	-0.02	-	-
	Asp92	0.60	-0.42	0.37	0.55	1.09	-0.59	-	-	-	-
	Ala59	0.43	0.08	-0.01	0.35	0.85	-0.47	-	-	-	-
G12C-Y96D-ADAGRASIB	Asp96	2.08	0.72	-0.70	1.32	3.42	-3.99	-	-	0.00	-
	Tyr64	1.52	-0.02	0.00	1.25	2.75	-3.76	-0.06	-	0.00	-
	His95	0.93	-0.27	0.14	0.76	1.55	-2.06	-	-	0.00	-
	Lys16	0.40	0.34	0.32	0.45	1.51	0.00	-	-	-0.93	-
	Arg102	0.47	-0.13	0.17	0.40	0.90	0.00	-	-	0.00	0.00
	Asp92	0.42	0.79	-0.82	0.38	0.77	-0.04	-	-	-	-
G12C-Y96D-MK-1084	Asp96	1.54	-13.37	13.06	1.42	2.65	-13.34	-0.11	-	0.00	-
	Gln99	1.32	0.73	-0.40	1.15	2.79	-3.20	-	-	0.00	-
	Arg68	1.10	14.41	-13.86	0.14	1.79	-0.02	-	-	0.07	0.00
	Glu63	1.01	-9.59	9.54	0.79	1.76	-0.23	-	0.00	-	-
	Asp92	0.48	-12.45	12.30	0.44	0.76	-0.42	-	-	-	-
	Thr58	-0.06	-0.99	1.60	0.08	0.64	-0.02	-	-	0.00	-

Supplementary Table S3 Key residue energy decomposition and delta interaction frequency for KRAS G12C-Y96S secondary mutant complex.

Mutant	Residue	Per Residue Decomposed binding free energy (kcal / mol)					Delta Interaction Frequency (Contact per Frame)				
		vdW	Ele.	Polar Solv.	Non-polar Solv.	Total	hydrophobic	π–π stack	H-Bond Donor	H-Bond Acceptor	π-cation
G12C-Y96S-SOTORASIB	Ser96	2.15	0.59	-0.19	1.78	4.33	-18.24	-0.53	-	-	-
	Als59	0.64	0.24	-0.16	0.62	1.34	-0.47	-	-	-	-
	Gly13	0.02	1.12	0.07	-0.01	1.20	-	-	-	-	-
	Als11	-0.08	1.14	-0.02	-0.09	0.95	-	-	-	-	-
	Lys16	0.37	0.59	-0.12	0.02	0.85	-	-	-	-0.04	-
	Gln99	0.29	0.10	-0.05	0.26	0.60	-1.43	-	0.00	-0.12	-
G12C-Y96S-ADAGRASIB	Ser96	2.19	0.06	-0.14	1.21	3.32	-5.72	-	-	0.00	-
	Arg68	0.53	-0.03	0.11	0.43	1.04	-0.78	-	-	-	-
	Glu62	0.31	0.43	-0.34	0.47	0.88	3.07	-	-	-	-
	Glu63	0.35	0.07	-0.07	0.19	0.54	0.00	-	-	-	-
G12C-Y96S-MK-1084	Ser96	2.38	-0.14	0.32	1.92	4.47	-14.21	-0.11	-	0.00	-
	Gln99	0.18	0.31	-0.22	0.24	0.51	-2.20	-	-	0.06	-

Supplementary Table S4 Key residue energy decomposition and delta interaction frequency for KRAS G12C-G13D secondary mutant complex.

Mutant	Residue	Per Residue Decomposed binding free energy (kcal / mol)					Delta Interaction Frequency (Contact per Frame)				
		vdW	Ele.	Polar Solv.	Non-polar Solv.	Total	hydrophobic	π–π stack	H-Bond Donor	H-Bond Acceptor	π-cation
G12C-G13D-SOTORASIB	Asp13	-0.45	3.62	-0.85	-0.46	1.86	-	-	-	-	-
	Arg68	0.83	-0.09	-0.06	0.66	1.35	0.00	-	0.00	0.07	-0.49
	Gln99	0.46	-0.02	0.05	0.39	0.88	-2.56	-	0.00	-0.10	-
	Glu63	0.39	-0.13	0.16	0.38	0.80	-0.37	-	-0.02	-	-
	Thr58	0.26	0.17	-0.12	0.40	0.70	-1.29	-	-	-0.01	-
G12C-G13D-ADAGRASIB	Asp13	-0.52	0.98	0.40	-0.28	0.57	-	-	-	-	-
	Arg68	0.92	-0.15	0.24	0.58	1.59	2.21	-	-	-	-
	Lys16	0.25	1.11	-0.24	0.39	1.51	0.00	-	-	-0.93	-
	Val9	0.59	0.04	0.00	0.46	1.10	-2.18	-	-	-	-
	Pro34	0.50	-0.04	0.02	0.41	0.89	-	-	-	-	-
	Glu62	0.26	0.50	-0.45	0.32	0.63	0.23	-	-	-	-
G12C-G13D-MK-1084	Asp13	-0.18	-14.03	12.38	-0.09	-1.92	-	-	-	-	-
	Lys16	-0.03	18.44	-16.10	0.58	2.89	0.00	-	-	-0.97	-
	Ala59	1.30	-0.50	0.57	1.15	2.52	-0.53	-	-	-	-
	Thr58	1.27	-0.65	0.45	0.96	2.04	-0.02	-	-	0.00	-

Supplementary Table S5 Key residue energy decomposition and delta interaction frequency for KRAS G12C-Q99L secondary mutant complex.

Mutant	Residue	Per Residue Decomposed binding free energy (kcal / mol)					Delta Interaction Frequency (Contact per Frame)				
		vdW	Ele.	Polar Solv.	Non-polar Solv.	Total	hydrophobic	π–π stack	H-Bond Donor	H-Bond Acceptor	π-cation
**G12C-Q99L-SOTORASIB**	Leu99	-0.07	0.08	-0.09	-0.10	-0.17	9.21	-	0.00	-0.12	-
	Gly13	0.03	1.25	0.07	0.00	1.36	-	-	-	-	-
	Ala11	-0.01	1.26	-0.04	-0.01	1.21	-	-	-	-	-
	Gln61	0.56	0.32	-0.28	0.51	1.12	0.00	-	-	0.00	-
	Lys16	0.34	0.72	-0.19	-0.05	0.81	-	-	-	0.00	-
**G12C-Q99L-ADAGRASIB**	Leu99	0.08	0.00	0.14	-0.28	-0.07	-3.05	-	-	0.00	-
	Glu62	2.13	-0.43	0.48	1.79	3.97	-1.26	-	-	-	-
	Tyr64	1.68	0.03	-0.04	1.27	2.94	-3.76	-0.06	-	0.00	-
	Met72	0.91	-0.02	0.03	0.67	1.60	-12.64	-	-	-	-
	Lys16	0.28	1.45	-0.50	0.36	1.59	0.00	-	-	-0.93	-
	Ile100	0.53	-0.02	0.06	0.63	1.20	-2.65	-	-	-	-
	Val9	0.67	-0.11	0.05	0.48	1.09	-2.17	-	-	-	-
**G12C-Q99L-MK-1084**	Leu99	0.00	0.31	-0.21	-0.25	-0.16	9.02	-	-	0.00	-
	Arg68	1.84	18.23	-16.94	0.66	3.79	-0.04	-	-	0.00	-0.28
	Glu63	1.46	-7.72	7.72	1.11	2.58	-0.58	-	0.00	-	-
	Ala59	0.61	0.07	0.18	0.44	1.30	-0.13	-	-	-	-
	Gly60	0.65	-1.85	1.79	0.49	1.08	-	-	-	0.00	-
